# Metabolic cell death networks in Alzheimer’s disease: mechanistic links and therapeutic perspectives of ferroptosis, cuproptosis, and disulfidptosis

**DOI:** 10.3389/fcell.2026.1879368

**Published:** 2026-07-30

**Authors:** Shuyu Ding, Zihan Guo, Hengyu Ji, Jinting He

**Affiliations:** Department of Neurology, China-Japan Union Hospital of Jilin University, Changchun, China

**Keywords:** Alzheimer’s disease, cuproptosis, disulfidptosis, ferroptosis, metabolic cell death

## Abstract

Alzheimer’s disease (AD) is a neurodegenerative disorder characterized primarily by progressive cognitive impairment, whose pathogenesis involves multiple pathological processes including protein deposition, metal homeostasis dysregulation, oxidative stress, mitochondrial dysfunction, and neuroinflammation. In recent years, metabolism-related cell death modalities such as ferroptosis, cuproptosis, and disulfidptosis have gradually been recognized as potentially involved in neuronal damage in Alzheimer’s disease. This review summarizes the fundamental mechanisms of ferroptosis, cuproptosis, and disulfidptosis, along with their research evidence in AD. Ferroptosis is primarily driven by iron imbalance, lipid peroxidation buildup, and impaired GPX4 defense. This process exhibits a bidirectional amplification loop with Aβ and tau pathologies. Cuproptosis contributes to neuronal damage through abnormal copper accumulation, FDX1-related mitochondrial protein lipoylation dysfunction, loss of iron-sulfur cluster proteins, and proteotoxic stress. Disulfidptosis links glucose metabolism disorders, insufficient reducing power, and actin cytoskeleton vulnerability, providing novel insights into metabolic stress and structural damage in AD. Furthermore, the three modes of cell death can undergo cross-regulation through the SLC7A11–NADPH–GSH/GPX4 axis, the FDX1–DLAT/DLST–iron-sulfur cluster axis, as well as upstream factors such as p53, NRF2, and AMPK. Metabolic cell death may constitute a critical pathological network in AD. Targeting these death pathways and their shared hubs is expected to provide new directions for disease stratification, biomarker development, and disease-modifying therapies.

## 1 Introduction

Alzheimer’s disease (AD) is a neurodegenerative disorder characterised primarily by progressive cognitive decline and is one of the most common causes of dementia in older adults. Clinically, patients with AD typically exhibit a gradual decline in cognitive abilities, impaired memory, compromised executive function and psychobehavioural abnormalities, ultimately leading to severe impairment in activities of daily living. As the global population ages at an accelerating rate, the number of people affected by AD continues to rise. Currently, over 50 million people worldwide are affected by AD, and it is projected that this figure will rise to more than 150 million by 2050, placing immense pressure on public health and socio-economic systems (Livingston et al., 2024; Porsteinsson et al., 2021; GBD 2016 Disease and Injury Incidence and Prevalence Collaborators, 2017). In-depth analysis of the pathogenesis of AD and the identification of new intervention targets have become key priorities in neurodegenerative disease research. From a pathological perspective, AD is characterised by two hallmark features: firstly, senile plaques formed by the deposition of extracellular β-amyloid (Aβ); and secondly, neurofibrillary tangles (NFTs) resulting from the aggregation of hyperphosphorylated tau protein within cells (Serrano-Pozo et al., 2011; Nelson et al., 2012). These pathological alterations contribute to synaptic dysfunction, neuroinflammation, and extensive neuronal loss, leading to cognitive decline (Guo et al., 2020; Tönnies and Trushina, 2017). Historically, research on AD has revolved around the “amyloid cascade hypothesis,” which suggests that the abnormal generation and accumulation of Aβ are critical initial triggers for the disease’s onset and progression, subsequently leading to a cascade of pathological events, including abnormal tau phosphorylation, oxidative stress and neuroinflammation (Hampel et al., 2021). Although various therapeutic strategies targeting Aβ have shown some promise in preclinical studies, their clinical translation has been largely limited for a long time. In recent years, the approval of anti-Aβ monoclonal antibodies such as lecanemab and donanemab has driven the development of disease-modifying therapies for Alzheimer’s disease. These drugs have demonstrated statistically significant clinical benefits in patients with early-stage AD, but their efficacy is still mainly reflected in slowing clinical progression, suggesting the complexity and multifactorial nature of the pathogenesis of AD (Almeida et al., 2025; van Dyck et al., 2023).

The degeneration of neurons is considered a major pathological factor contributing to cognitive decline in AD, with mechanisms of cell death being pivotal in this context (Guo et al., 2024). Recent advancements in cellular and molecular biology have revealed that neuronal loss is not limited to conventional apoptosis or necrosis; rather, it encompasses a variety of intricately regulated programmed cell death (RCD) pathways. Lately, alongside established cell death mechanisms like apoptosis, necrotic apoptosis, and pyroptosis, attention has shifted to a novel type of cell death primarily linked to metabolic disturbances, including ferroptosis, cuproptosis, and disulfidptosis (Song R. et al., 2025). Unlike traditional cell death forms, these emerging types are characterized by their unique metabolic dependencies, often associated with the disruption of cellular metabolic balance and involving critical processes such as imbalances in metal ion homeostasis, disturbances in the redox system, and compromised mitochondrial energy metabolism (Ercin et al., 2026). In recent years, this process has been extensively studied in neurodegenerative diseases (Alzheimer’s disease, Parkinson’s disease, amyotrophic lateral sclerosis, prion diseases), tumors, and other metabolic stress-related disorders, suggesting that it may represent a mode of metabolic cell death with cross-disease significance (Zefrei et al., 2024; Zhong et al., 2024; Zayed et al., 2026).

It is important to highlight that the forms of cell death associated with metabolism closely correspond to the pathological conditions present in the brains of AD patients. Studies reveal that brain tissue from AD patients shows not only an unusual buildup of metal ions like iron and copper but also significant metabolic disruptions, such as increased lipid peroxidation, an imbalance in antioxidant defenses, and compromised mitochondrial function (Quan et al., 2026; Tao et al., 2025). The excessive presence of iron ions can initiate ferroptosis by enhancing lipid peroxidation, which in turn worsens oxidative stress and neuronal injury (Quan et al., 2026). Additionally, disturbances in copper balance may lead to cuproptosis by interfering with the mitochondrial tricarboxylic acid (TCA) cycle and the clustering of acetylated proteins (Tao et al., 2025). The observed decline in glucose metabolism and energy-related disorders in the brains of those with AD may create an environment conducive to disulfidptosis (Gu Q. et al., 2024). These metabolic irregularities are not only critical pathological characteristics of AD but also serve as fundamental contributors to these emerging forms of cell death. Therefore, revisiting the molecular mechanisms underlying neuronal damage in AD through the lens of metabolism-related cell death could provide a fresh theoretical framework for comprehending the progression of the disease.

Recent studies indicate that different types of RCD are interconnected rather than occurring independently; they share common signaling pathways and essential molecules, showcasing unique aspects of cross-regulation and creating a highly intricate network for regulating cell death (Guo et al., 2024). Within this intricate system, critical elements such as redox balance, mitochondrial function, metal ion regulation, and inflammatory signaling can concurrently influence various cell death modalities and trigger distinct death programs in response to diverse pathological states. This is especially relevant in complex neurodegenerative disorders like AD, where metabolic pathways of cell death—including ferroptosis, cuproptosis, and disulfidptosis—are likely to interact through fundamental mechanisms such as oxidative stress, metal ion dysregulation, and energy metabolism disruptions, all of which contribute to neuronal injury and synaptic loss (Guo et al., 2024; Quan et al., 2026; Tao et al., 2025; Gu Q. et al., 2024; Xiao et al., 2026). Thus, understanding the integration of various metabolism-related cell death mechanisms is crucial for uncovering the molecular underpinnings of neurodegeneration in AD.

In light of this context, the present study seeks to transcend the conventional single-pathway research approach by offering a comprehensive review of advancements in the understanding of ferroptosis, cuproptosis, and disulfidptosis in the context of AD. Our focus is on elucidating the molecular mechanisms underlying these metabolism-associated cell death processes, while also gathering experimental data that highlights their involvement in AD. We delve into the potential interconnected molecular hubs and regulatory networks that link these pathways. Additionally, this article examines these cell death mechanisms to assess possible therapeutic approaches and their future clinical implications. By synthesizing current research insights, this review aims to reconstruct the molecular landscape of neuronal injury in AD through the lens of cell death networks, thereby offering theoretical foundations for the identification of new therapeutic targets and precision intervention strategies.

## 2 Ferroptosis

### 2.1 Mechanism of ferroptosis

Ferroptosis is a form of regulated cell death driven by iron-dependent lipid peroxidation, with core features including iron homeostasis imbalance, accumulation of membrane lipid peroxides, and impairment of the antioxidant defense system (Jiang et al., 2021; Xie et al., 2017). In AD, ferroptosis may intertwine with processes such as Aβ deposition, tau pathology, mitochondrial dysfunction, oxidative stress, and neuroinflammation. In the AD brain, especially in vulnerable regions such as the hippocampus and cortex (Wu et al., 2024), abnormal iron deposition and dysregulation of iron metabolism can occur. Excessive Fe^2+^ can promote ROS generation through the Fenton reaction and exacerbate oxidative damage to membrane lipids, thereby providing a pathological basis for ferroptosis (Jiang et al., 2021; Tan et al., 2021).

Lipid peroxidation is a key execution step of ferroptosis. Neuronal, synaptic, and mitochondrial membranes rich in polyunsaturated fatty acids (PUFAs) are susceptible to free radical attack (Kagan et al., 2017); lipid metabolic enzymes such as ACSL4 and LPCAT3 promote the incorporation of PUFAs into membrane phospholipids, forming a pool of easily oxidizable substrates (Jiang et al., 2021; Pope and Dixon, 2023). In the context of AD-related iron accumulation, mitochondrial dysfunction, and chronic oxidative stress, lipid hydroperoxides continuously accumulate, which can compromise membrane integrity and exacerbate neuronal damage (Fujii and Yamada, 2023; Stoyanovsky et al., 2019).

Decreased anti-lipid peroxidation defense further increases cellular susceptibility to ferroptosis. System Xc^−^ supports the synthesis of cysteine and glutathione (GSH) by importing extracellular cystine, while GPX4, which relies on GSH to detoxify toxic lipid peroxides, serves as the core defense against ferroptosis. When the function of System Xc^−^ is impaired, GSH is depleted, GPX4 activity declines, or NADPH supply is insufficient, the ability to clear lipid peroxides is diminished (Li F. J. et al., 2022; Yang et al., 2014; Dixon et al., 2012). Nrf2 can maintain anti-ferroptosis defense by regulating SLC7A11, GSH synthesis, and the expression of multiple antioxidant genes (Anandhan et al., 2020; Song and Long, 2020); the FSP1/CoQ10 axis, as a GPX4-independent supplementary defense line, participates in limiting the amplification of lipid peroxidation (Bersuker et al., 2019; Doll et al., 2019). Therefore, AD-related ferroptosis can be understood as a result of the combined effects of iron deposition, lipid peroxidation, mitochondrial damage, and antioxidant defense exhaustion.

### 2.2 Evidence and pathological networks of ferroptosis in AD

#### 2.2.1 Direct evidence from human brains and AD models

Human AD brains exhibit multiple ferroptosis-associated alterations, including iron dyshomeostasis, elevated lipid peroxidation, and compromised antioxidant defenses (Qiang et al., 2024). Post-mortem studies reveal extensive iron accumulation in the hippocampus and cerebral cortex, which strongly correlates with cognitive decline (Ma et al., 2022).

Thorwal et al. (2025), after rigorously excluding residual blood contamination, confirmed increased iron deposits in the prefrontal cortex of AD patients, along with elevated 4-HNE and 3-nitrotyrosine levels. These findings provide direct evidence of iron-related oxidative damage in the human AD brain.

Complementary evidence from animal models further supports this link. Wu et al. (2024) used a DNAzyme-based fluorescent sensor to detect an abnormal Fe^3+^/Fe^2+^ ratio near Aβ plaques in AD model mice, revealing a profound redox imbalance in the plaque microenvironment.

These findings lend strong support to the notion that iron-related oxidative damage occurs in the brain. By integrating these histological findings with evidence of oxidative harm, it can be concluded that the unusual iron accumulation in the brain has a robust pathological foundation; further analysis indicates that this anomaly likely stems from a breakdown in the finely-tuned regulatory mechanisms governing iron uptake, storage, and release. In research focused on iron metabolism regulatory proteins, Bao and his team discovered that levels of the mammalian-specific non-heme iron efflux protein (FPN) are significantly lower in both human brain tissue and animal models. Moreover, the deletion of the FPN gene results in hippocampal shrinkage, deficits in learning and memory, and a range of changes akin to ferroptosis, such as reduced mitochondrial size, elevated lipid peroxides, and compromised GPX4-related protective functions (Bao et al., 2021). This implies that the disruption of iron efflux pathways may be a crucial factor contributing to ferroptosis-like damage in the brain.

Alongside disruptions in iron regulation, there is a significant rise in lipid peroxidation observed in the brains of AD patients. A study conducted by Park et al. reveals that the concentrations of lipid peroxidation byproducts, including 4-HNE and malondialdehyde (MDA), are heightened in both the brain tissues of AD patients and corresponding animal models (Park et al., 2021). This suggests that oxidative harm to membrane lipids rich in PUFAs intensifies as the condition advances (Park et al., 2021). Additionally, it is crucial to recognize that the antioxidant defense mechanism involving System Xc^−^–GSH–GPX4 in the brains of these patients is compromised. This impairment leads to a notable decrease in the cells’ capacity to eliminate lipid peroxides, thereby creating favorable biochemical conditions for the onset of ferroptosis (Chen et al., 2021).

#### 2.2.2 Bidirectional coupling between ferroptosis and Aβ/tau pathology

Studies reveal that ferroptosis and the two key pathological characteristics of AD—the development of Aβ plaques and tau tangles—are interconnected through a reciprocal pathological relationship rather than a one-way link. Excess iron leads to irregular processing of amyloid precursor protein (APP), which in turn accelerates Aβ production. Research has shown that increased levels of intracellular iron diminish the transcription of the Furin gene while boosting β-secretase activity, thus facilitating the conversion of APP into Aβ40/42 (Wang et al., 2022). On the flip side, Aβ can trigger ferroptosis. In the brain tissue of AD individuals, the pathological alterations related to Aβ in the grey matter during the later stages of the disease are often associated with heightened iron levels, increased expression of NCOA4, and decreased GPX4 expression. This indicates that Aβ might worsen neuronal ferroptosis by promoting ferritin autophagy while hindering the cell’s capacity to eliminate lipid peroxides. However, these alterations are less pronounced in the cerebral white matter or the occipito-temporal cortex, implying that the ferroptosis pathology differs across various brain regions and stages of AD (Majerníková et al., 2024). Additionally, there is a significant interplay between tau pathology and ferroptosis. Mayr et al. observed in their examination of human brain tissue that the expression of ferritin in TfR-positive degenerating neurons and microglia rises with the advancement of NFTs; furthermore, abnormal expression of genes related to ferroptosis can be identified even in the pre-tangled phase and in certain p-tau-negative neurons, suggesting that disruptions in iron metabolism and ferroptosis signaling may occur before the complete development of typical tau tangles and contribute to the neuronal degeneration process characterized by droplet-like degeneration (DD) (Mayr et al., 2024). More direct mechanistic investigations also support a synergistic relationship between tau and ferroptosis. In studies primarily conducted on animal models, An et al. discovered that lactylation at the tau K677 site leads to the degradation of FTH1 and increases free iron levels by modulating NCOA4-mediated ferritin autophagy, thereby intensifying ferroptosis and worsening cognitive decline (An et al., 2024). Conversely, in cellular model studies, investigations into small molecules such as dopamine-type polyphenol (PDP), levodopa-type polyphenol (PLDP), and the tryptophan derivative GCTR (a hybrid small molecule synthesized from valeric acid and L-tryptophan) have demonstrated that both tau liquid-liquid phase separation and ferroptosis can be concurrently influenced. Specifically, PDP and PLDP inhibit ferroptosis by chelating free iron while also reducing tau liquid-liquid phase separation and aggregation; GCTR, on the other hand, restores GPX4 activity, mitigates ferroptosis, and simultaneously intervenes in Fe^3+^-induced tau liquid-liquid phase separation and abnormal fibrillation (Moorthy et al., 2024).

**FIGURE 1:**
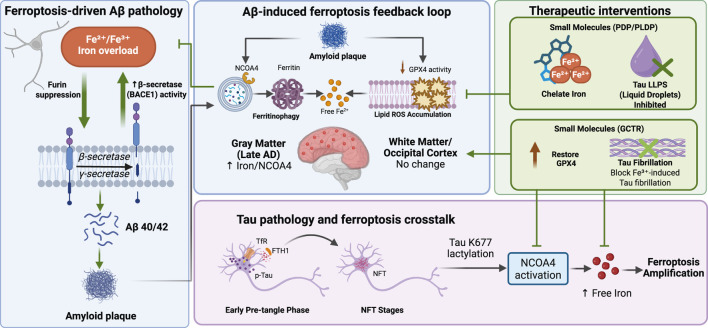
Bidirectional coupling of ferroptosis and Aß/Tau pathology in Alzheimer’s disease. Ferroptosis may participate in the positive feedback loop involving Aβ/Tau pathology, oxidative damage, and neuronal degeneration across different brain regions and disease stages in AD. Iron overload can promote the generation of Aβ40/42 and the formation of amyloid plaques by inhibiting Furin expression and enhancing abnormal processing of β/γ-secretase-associated APP. Conversely, Aβ deposition can also induce NCOA4-mediated ferritinophagy, free iron release, decreased GPX4 activity, and accumulation of lipid ROS, thereby amplifying neuronal ferroptosis. There is also an interaction between tau pathology and ferroptosis. Abnormalities in TfR, ferritin, and ferroptosis-related signaling suggest that iron metabolism dysregulation may be involved in the process from the pretangle stage to NFT formation. Tau K677 lactylation can further enhance ferroptosis by activating NCOA4, promoting FTH1 degradation, and increasing free iron. Therapeutically, small molecules such as PDP/PLDP can chelate Fe^2+^/Fe^3+^ and inhibit tau liquid-liquid phase separation, while GCTR can restore GPX4 activity and block Fe^3+^-induced tau fibrillation.

Current evidence indicates that ferroptosis in AD is not simply a consequence of Aβ/tau pathology. This process may serve varying functions throughout the progression of the disease: in its initial phases, it is characterized by an imbalance in iron metabolism and heightened vulnerability to ferroptosis, while in the later phases, it functions as a crucial mechanism that exacerbates protein aggregation and neuronal injury. Although the majority of research supports the notion of ferroptosis-related pathology in AD, it remains unverified whether GPX4 is significantly reduced across all brain regions in the cortex (Majerníková et al., 2024), implying that ferroptosis might only be relevant in certain areas of the brain or at specific stages of the disease (Figure 1).

#### 2.2.3 Glial cells and neurovascular units: an amplification loop

Ferroptosis associated with AD extends beyond just the damage to neurons; it is intricately woven into a pathological framework that includes glial cells and neurovascular units (NVU), perpetuating a harmful cycle. Cai et al. (2025) found in an animal model with microglia-specific deletion of the Atg7 gene that Atg7 deficiency impairs the ability of microglia to aggregate and engulf Aβ plaques, leading to more pronounced plaque spreading and exacerbating plaque-associated neuritic damage. Mechanistically, Atg7 deficiency is accompanied by reduced UPR, increased oxidative stress, and elevated lipid peroxidation, making microglia more susceptible to ferroptosis-related changes under Aβ proteotoxic stress. However, it should be noted that autophagy deficiency may also sensitize cells to other types of regulated cell death, such as apoptosis and necroptosis. Therefore, in this model, the relative contributions of ferroptosis and other cell death pathways remain to be further investigated (Cai et al., 2025). On the other hand, certain microglial signaling pathways can mitigate damage related to ferroptosis. For example, studies involving both animal and cellular models have shown that exosomes from M2 microglia can transport triggering receptor expressed on myeloid cells 2 (TREM2) protein, which activates the Wnt/β-catenin signaling pathway, thereby reducing Aβ-induced neuronal ferroptosis, inflammation, and oxidative stress in mouse models (Zhu et al., 2025). At the level of astrocytes, the lack of Nrf2 and the increase of NADPH oxidase 4 (NOX4) are seen as critical connections between oxidative stress and ferroptosis. Research led by Tang et al. (2024) revealed that in the frontal cortex of AD patients and in 3×Tg mouse models, Nrf2 levels were diminished while NOX4 levels were elevated. Additionally, the absence of Nrf2 resulted in decreased expression of heme oxygenase-1 (HO-1) and GPX4, along with increased ROS levels, worsened lipid peroxidation, and mitochondrial fragmentation. This indicates that impairment of the astrocytic antioxidant defense may worsen ferroptosis-related damage in AD (Tang et al., 2024). Further single-cell transcriptomic studies and both *in vitro* and *in vivo* experiments have shown that NOX4 is significantly expressed in astrocytes affected by AD, and reducing NOX4 expression can alleviate ferroptosis in these cells, leading to improved cognitive function and lower levels of Aβ and p-tau. These results imply that astrocytic ferroptosis could be a crucial mechanism linking metabolic oxidative stress to the fundamental proteopathic processes of AD (Maimaiti et al., 2024).

Ferroptosis, alongside glial cells, plays a significant role in the advancement of AD by impairing the NVU. Research indicates that in the cerebral cortex of AD individuals, there is a synergistic increase in proprotein convertase subtilisin/kexin type 9 (PCSK9) and Aβ within the blood-brain barrier (BBB) area, which is associated with heightened iron accumulation and the activation of the ferroptosis pathway, particularly affecting brain microvascular endothelial cells (BMECs). Additionally, studies involving high-fat diet-induced apolipoprotein E knockout (ApoE^−/−^) mice and *in vitro* BBB models using HCMEC/D3 cells have shown that ferroptosis in BMECs mediated by PCSK9 directly undermines the integrity of the BBB (Zhang H. et al., 2026). Moreover, post-mortem analyses of brain tissue from AD patients have revealed collagen buildup in small veins and alterations in the extracellular matrix, where increased agrin levels can trigger ferroptosis in pericytes. This process results in diminished expression of platelet-derived growth factor receptor β (PDGFRβ) and the tight junction protein claudin-5, further destabilizing the BBB (Cao et al., 2025a). In addition to vascular matrix remodeling, Aβ itself may also directly participate in pericyte injury and affect BBB stability. In APP/PS1 transgenic mouse models and Aβ1-40 *in vivo* treatment models, researchers found that Aβ1-40 can be taken up by pericytes via the CD36 receptor, inducing mitochondrial damage and PINK1/Parkin-related mitophagy, accompanied by Fe^2+^ accumulation, elevated lipid ROS, and ferroptosis-related changes such as decreased GPX4, xCT, and ferritin. This suggests that Aβ may increase pericyte vulnerability through mitochondrial stress and ferroptosis-related oxidative damage, ultimately impairing pericyte function and disrupting BBB integrity (Li J. et al., 2022). These findings imply that mitochondrial autophagy-related ferroptosis in pericytes may be a crucial mechanism connecting Aβ-induced vascular damage to the NVU’s impairment.

In conclusion, ferroptosis can take place in different cell types such as microglia, astrocytes, BMECs, and pericytes, creating a detrimental cycle characterized by increased inflammation, disruption of the BBB, and reduced clearance of Aβ.

#### 2.2.4 Mitochondrial, ER, and metabolic abnormalities increase ferroptosis susceptibility

In individuals with AD, numerous metabolic irregularities and dysfunctions in organelles can greatly diminish the resilience of neurons to ferroptosis, leading to an increased vulnerability to this process. The impairment of mitochondrial function and energy metabolism restricts the availability of cellular reducing agents. A proteomic analysis involving 625 samples from the inferior temporal cortex revealed that as the condition advances, there is a notable reduction in mitochondrial proteins, which coincides with a drop in GSH levels. Since the production of GSH relies on ATP availability, a lack of mitochondrial energy hampers the replenishment of GSH, thus heightening the risk of ferroptosis in cells (Alves et al., 2025).

Endoplasmic reticulum (ER) stress and disturbances in calcium homeostasis are significant factors that trigger ferroptosis in AD. Research has shown that orosomucoid-like 3 (ORMDL3) levels are elevated in the serum of AD individuals, as well as in the brain tissue of Appswe/PS1dE9 mice and BV2 cell models. This protein contributes to oxidative stress and ferroptosis through the PERK/ATF4/HSPA5 pathway, indicating that ER stress may hasten the development of AD-like symptoms in a neuroinflammatory context (Shao et al., 2023). Additionally, in neuron-like cell models induced by Aβ1-42, an increase in ANO6 protein expression was noted; this protein modulates ER stress-related ferroptosis via the TMEM30A/PERK–eIF2α–ATF4–CHOP signaling pathway, implying that Aβ-induced neuronal injury could be partly driven by this mechanism (W et al., 2025). From a treatment standpoint, in induced pluripotent stem cell (iPSC) models from ApoE4/E4 sporadic AD patients, lithium chloride not only lowers cytoplasmic Fe^2+^ levels and decreases DMT1 expression but also enhances GPX4 activity, reduces lipid peroxidation, and mitigates ROS buildup. Moreover, it improves mitochondrial respiratory function and suppresses the expression of the ER calcium channel InsP3R-1. These results indicate that disturbances in ER-Ca^2+^/mitochondrial balance may be closely associated with increased ferroptosis risk in AD (Wang et al., 2026). Furthermore, metabolic disorders and issues with lipid metabolism also heighten the likelihood of ferroptosis. A high-fat diet worsens cognitive decline, Aβ accumulation, and inadequate m6A modification in AD animal models, while simultaneously increasing the expression of insulin-like growth factor 2 mRNA-binding protein 2 (IGF2BP2). In follow-up intervention studies, silencing IGF2BP2 restored SLC7A11 expression and reduced oxidative stress linked to ferroptosis, an effect similar to that of the ferroptosis inhibitor Fer-1, suggesting that metabolic irregularities may amplify susceptibility to ferroptosis through a dual mechanism involving epigenetic changes and the antioxidant defense system (Zhang D. et al., 2026).

#### 2.2.5 RNA/epigenetic regulation and multi-omics clues

In recent times, irregularities in RNA regulation have emerged as a significant upstream element connecting AD to ferroptosis. Studies on AD models indicate that the levels of specific miRNAs decline and are inversely related to the heightened expression of IGFBP-2 and ferritin accumulation in neurons. Experimental techniques, including the use of miRNA mimics or inhibitors, dual-luciferase reporter assays, and RNA immunoprecipitation, have validated that these miRNAs may influence the expression of IGFBP-2 and ferritin, thereby affecting the Nrf2/SLC7A11/GPX4 signaling pathway and ultimately modifying antioxidant enzyme activity and oxidative stress. These results imply that the miRNA–IGFBP-2–ferritin pathway could play a role in the relationship between iron metabolism disturbances and ferroptosis in AD (Luo et al., 2025). Additionally, research by Gu X. et al. (2024) in a scopolamine-treated mouse model and an Aβ1-42-induced SH-SY5Y cell model revealed that methyltransferase-like protein 14 (METTL14) can inhibit the expression of taurine-upregulated gene 1 (TUG1) through m6A modification, which in turn diminishes TUG1’s suppressive effect on the growth differentiation factor 15 (GDF15)/NRF2 signaling pathway, ultimately reducing ferroptosis (Gu X. et al., 2024). This discovery indicates that epigenetic irregularities are likely to be a crucial upstream regulatory factor in ferroptosis associated with AD.

In addition to the involvement of specific miRNAs in the regulation of ferroptosis, the overall role of non-coding RNAs (ncRNAs) in AD has also attracted increasing attention. Among them, miR-107 can reduce Aβ levels by targeting BACE1, while miR-346 may increase Aβ production through modulating APP mRNA. Due to the high stability of certain ncRNAs in cerebrospinal fluid and peripheral blood, they not only hold potential as early biomarkers for AD but may also constitute an important regulatory network connecting multiple pathological signals and metabolic cell death pathways (Tripathi et al., 2024).

Research into functional RNAs, alongside multi-omics and bioinformatics studies, has revealed iron-dependent cell death-related molecular networks in AD on a systemic scale. Transcriptomic signatures from TrioSig and the integrated stress response mediated by eIF2α-ATF4 are notably enriched in both AD mouse models and human brain samples, suggesting a potential link between oxidative stress-related cell death, or ferroptosis, and its upstream stress pathways in the disease’s progression (Currais et al., 2025). However, these findings are largely derived from signature enrichment analyses and *in vitro* studies. In a similar vein, Liu et al. (2026) discovered nine genes linked to ferroptosis through various public datasets and established a regulatory network involving miRNAs and ferroptosis-related differentially expressed genes (FRDEGs). This research implies that the irregular expression of these genes in peripheral blood or the brain may be co-regulated by upstream non-coding RNAs, although it primarily relies on correlational data (Liu et al., 2026). Additionally, molecules like peroxiredoxin 6 (PRDX6) and ACSL1 have emerged as potential targets within astrocyte networks and the interplay between immunometabolism and ferroptosis at the BBB (Li et al., 2026; Miao et al., 2025). Overall, current RNA and multi-omics investigations indicate that ferroptosis in AD is not solely evident in the later stages of lipid peroxidation and membrane damage, but is also influenced by non-coding RNAs, m6A modifications, and systemic transcriptional network alterations. This body of work is still largely focused on exploring mechanisms and identifying candidate biomarkers, with the need for further validation of its causal relationships and clinical applicability.

In conclusion, ferroptosis is intricately involved in the complex and evolving pathology of AD. Research across human brain tissues, animal studies, and cellular models has consistently highlighted key pathological aspects of ferroptosis in AD, such as disruptions in iron balance, the buildup of lipid peroxidation, and the failure of the antioxidant defense system. Notably, ferroptosis does not occur in a vacuum during the advanced stages of neuronal injury; instead, it interacts in a reciprocal and detrimental cycle with Aβ and tau pathologies. This process is prevalent in microglia, astrocytes, and the NVU, creating a feedback loop within a network of different cell types. Additionally, mitochondrial dysfunction, ER stress, and metabolic imbalances further increase the susceptibility of neurons to ferroptosis. Recent findings regarding RNA and epigenetic regulatory mechanisms offer a more profound molecular understanding of this phenomenon. While much of the current data is primarily correlational, it is evident that ferroptosis acts as a crucial link among various pathological processes in AD, suggesting that targeting its regulatory pathways could lead to innovative treatment approaches for the disease.

### 2.3 Therapeutic strategies targeting ferroptosis in AD

#### 2.3.1 Targeting core execution mechanisms

Iron chelators are among the earliest therapeutic strategies used to intervene in iron homeostasis abnormalities. Deferoxamine (DFO) and deferiprone (DFP) reduce the risk of Fenton reaction and lipid peroxidation by chelating excess free iron ions within cells (Wang et al., 2023a; Ayton et al., 2025), which is mechanistically related to ferroptosis inhibition. Early small-scale clinical explorations suggested that DFO might slow cognitive decline in AD patients, and animal experiments have also shown that it can improve AD-related pathological manifestations (McLachlan et al., 1993; Kosyakovsky et al., 2021; Zhang et al., 2020). However, it should be emphasized that these early clinical studies were conducted before ferroptosis was defined as a form of regulated cell death, and therefore cannot be directly equated with modern clinical validation of targeting ferroptosis in AD treatment. In contrast, recent randomized clinical trials of DFP have shown that although DFP has good oral bioavailability and can reduce iron load in some brain regions, it did not show cognitive benefit in patients with early AD, and was instead associated with worsened cognitive decline. This result suggests that there is still significant uncertainty regarding the efficacy and safety of broad-spectrum iron chelation therapy.

Besides iron chelators, another crucial approach to prevent ferroptosis involves inhibiting lipid peroxidation. Ferroptosis inhibitor-1 (Fer-1) protects cells by neutralizing lipid radicals and interrupting the cascade of membrane lipid peroxidation reactions (Zilka et al., 2017; Skouta et al., 2014). In human AD brain organoid models, Fer-1 treatment has been shown to decrease amyloid accumulation, reduce 4-HNE levels, and improve iron storage issues, suggesting its potential to inhibit lipid peroxidation during the early stages of AD pathology (Majerníková et al., 2024). Additionally, fat-soluble antioxidants like vitamin E (α-tocopherol) have demonstrated anti-ferroptotic properties (Hu et al., 2021).

#### 2.3.2 Targeting upstream regulatory networks and metabolic pathways

In AD research, intervening in neuronal ferroptosis is considered a highly promising disease-modifying strategy. To achieve this goal, researchers have delved into the level of upstream regulatory networks and metabolic pathways to conduct extensive exploration, ultimately identifying numerous intervention directions with clear mechanisms and well-defined targets. The table above summarizes specific therapeutic approaches targeting upstream regulatory networks and metabolic pathways (Table 1).

**TABLE 1:** Therapeutic strategies targeting upstream regulatory networks and metabolic pathways of ferroptosis in Alzheimer’s disease.

Intervention strategy	Representative molecules/interventions	Core mechanism	Primary effects	References
Targeting the Nrf2 axis	Artemisinin, Ganoderic acid A (GAA), Astragalosides; Quercetin (QE)	Activation of the Nrf2–SLC7A11–GPX4 antioxidant network;	Reverses Aβ-induced ferroptosis and lipid peroxidation;	Deng et al. (2025), Lu Q. et al. (2025), Wang et al. (2025a), Liu et al. (2025)
	Polygala Compound (PCF), Yangming Kaixin Yizhi Formula (YKY);	Reshaping the expression of iron metabolism-related molecules (TFR1, FTH1, NCOA4, FPN1)	Alleviating hippocampal neuron loss;	Qian et al. (2026), Xiong et al. (2026a)
	Mao-Rui isoflavones, Forsythoside A, Anemarrhenin, Acorus tatarinowii, Schisandrin, Polygonum aviculare glycosides, etc.	​	Improved cognitive function in AD mice	Li et al. (2025), Li et al. (2024), Peng et al. (2025), Xiong et al. (2026b), Meng et al. (2025)
GPX4 activators	Thonningianin A (ThA), tannic acid (TA);	Directly bind to GPX4 and enhance its enzymatic activity;	Reduces ROS levels, lipid peroxidation and iron accumulation;	Yong Y. et al. (2024), Baruah et al. (2023)
	Penthorum chinense Pursh extract, Curculigoside	Upregulates GPX4 expression and increases the GSH/GSSG ratio	Alleviates Aβ/Tau-related toxicity and mitochondrial damage	Yong Y. Y. et al. (2024), Gong et al. (2024)
PI3K/Akt–NADK/NADPH axis	Exogenous Neuritin	Activates the PI3K/Akt signalling pathway;	Enhances neuronal reducing capacity and redox homeostasis;	Song D. et al. (2025)
		Enhances NADK activity and promotes NADPH production	Improves cognitive function and inhibits ferroptosis	​
Corrects organelle dysfunction and metabolic imbalances	Lithium chloride;	Improves mitochondrial respiratory function;	Alleviates organelle dysfunction and oxidative damage;	Wang et al. (2026)
	Targeting ORMDL3	Downregulates InsP3R-1, correcting dysfunction of the endoplasmic reticulum–Ca^2+^–mitochondrial axis;	Inhibits AD-associated ferroptosis pathology	Han et al. (2026)
	​	Inhibits PERK/ATF4/HSPA5 pathway-mediated endoplasmic reticulum stress	​	​
Towards lipid metabolism remodelling: gut microbiota metabolites	*Bacteroides* ovatus;	LPC activates GPR119, inhibiting ACSL4 expression	Alleviates lipid peroxidation-driven ferroptosis;	Zha et al. (2025)
	Lysosomal phosphatidylcholine (LPC)		Reduces Aβ burden and improves cognitive function	Zha et al. (2025)

In addition to natural products, synthetic small molecules based on rational drug design also provide new candidate directions for multi-target intervention in AD. Recent computer simulation studies have shown that 6-chlorocoumarin-oxadiazole hybrids and N-acetyl-1,3,4-oxadiazoline derivatives exhibit strong binding affinity for AD-related targets such as AChE, BuChE, and BACE-1, and demonstrate favorable predicted blood-brain barrier permeability and low toxicity characteristics (Shrivastava et al., 2025; Prabakaran et al., 2025). Although these studies are currently primarily at the stage of computational pharmacology and *in vitro* prediction, and cannot yet directly prove anti-ferroptotic effects, their multitarget profile suggests that future synthetic small-molecule drugs may indirectly influence susceptibility to metabolic cell death in the AD brain by simultaneously modulating pathways related to cholinergic dysfunction, Aβ generation, and oxidative stress.

#### 2.3.3 Targeting cell–cell interactions

##### 2.3.3.1 Glial–neuronal interactions

Recently, targeting microglial activation and its inflammation–ferroptosis crosstalk has become a key direction in AD therapy. The NLRP3–Nrf2 axis and BMP6/SMAD1 pathway are emerging as crucial regulatory hubs. These hubs link neuroinflammation, ferroptosis, and neuronal degeneration (Qiu et al., 2023). Semaglutide and ghrelin regulate these two signaling pathways respectively, promoting the conversion of microglia from the pro-inflammatory M1 phenotype to the protective M2 phenotype. This not only reduces the release of pro-inflammatory cytokines and ROS but also lowers the risk of cells undergoing ferroptosis (Zheng et al., 2026; Guo et al., 2025). Based on these findings, it can be inferred that microglia are highly likely to play a crucial role in cerebral inflammatory imbalance, the exacerbation of ferroptosis, and secondary neuronal damage. Comprehensive regulation of microglial inflammatory activation, phenotypic conversion, and susceptibility to ferroptosis holds promise as a novel disease-modifying therapeutic strategy for AD.

##### 2.3.3.2 Neurovascular unit and blood–brain barrier

In cortical cells of AD patients, elevated PCSK9 levels occur concurrently with ferrocytosis in BMECs and damage to the BBB (Mazura et al., 2022). Recent studies have shown that the PCSK9-targeting siRNA drug Inclisiran can inhibit PCSK9-mediated endothelial cell ferrocytosis by reducing PCSK9 expression in the brain, particularly in BMECs, thereby improving the structural and functional homeostasis of the BBB. Simultaneously, this drug is also highly effective in alleviating intracerebral accumulation caused by impaired Aβ clearance across the BBB, thereby reducing AD-like pathological changes (Zhang H. et al., 2026). These findings suggest that the PCSK9-ferroptosis-BBB axis is likely a key mechanistic link connecting lipid metabolism disorders, cerebral vascular endothelial damage, and amyloid pathological accumulation. Furthermore, this research provides new insights into disease-modifying treatments for AD centered on BBB protection.

##### 2.3.3.3 Combined intervention against Aβ/tau pathology and ferroptosis

Multi-tiered regulation of stress networks associated with AD proteinopathies is gradually becoming a key focus of research. Existing studies indicate that such interventions are not merely used to reduce Aβ deposition or abnormal tau modification; they can also simultaneously reduce neuronal susceptibility to ferroptosis by reshaping oxidative stress, inflammatory amplification, and lipid peroxidation networks. In particular, the combination of the histone deacetylase 6 (HDAC6) inhibitor WY118 with lithium chloride (LiCl) demonstrates a synergistic regulatory effect on the tau pathology–stress kinase–ferroptosis axis. This regimen inhibits the activity of HDAC6 and glycogen synthase kinase-3β (GSK-3β), enhancing α-tubulin acetylation, significantly reducing abnormal phosphorylation of tau at Ser396 and Thr231, and inhibiting stress signals such as p38 mitogen-activated protein kinase (p38 MAPK), while simultaneously restoring the expression of anti-ferroptosis molecules such as SLC7A11, thereby alleviating tau-related cytoskeletal damage and enhancing the neurons’ resistance to ferroptosis (Lu Z. et al., 2025). Notably, GSK-3β is not only a critical upstream kinase for Tau hyperphosphorylation but also participates in multiple pathological processes, including Aβ production, oxidative stress, and neuronal injury, thus potentially serving as a key regulatory node connecting Aβ/Tau pathology and ferroptosis susceptibility. Based on this mechanistic link, ergothioneine (ERGO) has been found to inhibit Aβ oligomer-induced hyperphosphorylation of Tau at Ser396 and reduce the expression of APP, BACE1, and nicastrin, an effect likely associated with GSK-3β inactivation. This suggests that inhibiting GSK-3β while simultaneously alleviating Aβ-related processing abnormalities and Tau hyperphosphorylation may indirectly attenuate oxidative stress and lipid peroxidation accumulation, thereby reducing the susceptibility of neurons to ferroptosis. Furthermore, *in silico* studies have identified natural compounds from Bryophyllum pinnatum, such as kaempferol and quercetin (Shibagaki et al., 2024; Babalola et al., 2024), as potential GSK-3β inhibitors, providing a theoretical basis for developing multi-target candidate compounds with combined anti-Tau pathology, anti-oxidative stress, and anti-ferroptosis potential.

The combined use of morin hydrate (MH), clinoptilolite (ZC), and physical and mental activities (PhM) (Abu-Elfotuh et al., 2025), as well as the bifunctional compound 21d (Lv et al., 2025), the multifunctional small molecule AC5 (Kumar et al., 2026), and berberine (BBR) (Li et al., 2023), have all demonstrated synergistic improvements in Aβ/tau-related pathology and ferroptosis-associated damage.

Among these, 21d addresses abnormalities associated with APP while also covering tau pathology and the GSH/GPX4 axis of iron metabolism; AC5 and BBR, on the other hand, place greater emphasis on the joint regulation of the cross-network linking Aβ-related protein toxicity, oxidative inflammatory responses, and ferroptosis. Overall, these findings suggest that multi-target intervention is not merely a matter of inhibiting a single pathological pathway, but rather involves the simultaneous correction of protein misfolding, dysregulation of stress kinases, neuroinflammation, and susceptibility to ferroptosis, thereby demonstrating the potential value of multi-target disease modification in delaying the progression of AD.

Despite the significant synergistic effects demonstrated by these multi-target natural products and their combinations in preclinical models, we must recognize their inherent limitations: many of the currently reported compounds are derived from natural products or natural product-like structural scaffolds, and most of the evidence is confined to *in silico* screening, cellular assays, or animal model experiments. Their clinical application remains constrained by pharmacological limitations, including low blood-brain barrier penetration, poor oral bioavailability, rapid metabolism, insufficient target specificity, unclear dose-response relationships, and limited long-term safety data. Therefore, future research should focus on optimizing structure-activity relationships, developing novel formulations (e.g., nanoparticles), or synthesizing analogs with improved drug-like properties to enhance their pharmacokinetic characteristics and translational potential.

##### 2.3.3.4 Novel delivery platforms and translational strategies

Novel delivery platforms are gradually emerging as a key translational direction for interventions targeting ferroptosis in AD. Their value lies not only in enhancing the efficiency of drug delivery to the brain but also in their ability to cross the BBB and accumulate at the site of the lesion, thereby synergistically regulating ferroptosis during the pathological process of AD through multifunctional integration. Currently, several delivery systems have demonstrated significant application potential. For instance, small extracellular vesicles extracted from milk can effectively transport miRNA-34, thereby alleviating oxidative stress and neuroinflammation in Aβ-induced mouse models, while modulating the expression of proteins associated with ferroptosis and mitochondrial function (Çelik et al., 2026); BBB-targeted biselenide nanospheres (CLNDSe NPs) inhibit ferroptosis by suppressing Aβ aggregation, alleviating oxidative damage, and restoring GPX4-related antioxidant defenses (Wang et al., 2023b); the brain/mitochondria dual-targeting smart nanomedicine TQCN alleviates neuronal damage caused by iron overload and lipid peroxidation through multi-level intervention combining iron chelation, inhibition of free radical burst, and reconstruction of the endogenous antioxidant defense system (Liu et al., 2024).

In addition, biological delivery strategies based on cell-derived exosomes also show promise. MSC-Exos can carry functional cargoes such as miRNAs, proteins, lipids, and non-coding RNAs, modulating ROS production, GSH metabolism, lipid peroxidation, and anti-ferroptosis-related pathways including Nrf2 and SLC7A11/GPX4, thereby alleviating oxidative stress and ferroptosis-related damage (Zayed et al., 2025). Overall, delivery systems with brain-targeting capability, multifunctional integration, and biocompatibility hold significant potential as a translational approach for targeted ferroptosis intervention in AD.

These findings suggest that delivery systems with brain-targeting properties and multifunctional integration capabilities hold promise as a key translational direction for disease-modifying treatments in AD.

##### 2.3.3.5 Barriers to clinical translation

Interventional strategies targeting ferroptosis-related damage in AD have progressively expanded from early iron homeostasis regulation and anti-lipid peroxidation to include modulation of the Nrf2–SLC7A11–GPX4 axis, restoration of NADPH reducing power, protection of organelle homeostasis, glial cell regulation, and brain-targeted delivery. However, their clinical translation still faces significant limitations.

First, research on traditional iron chelators cannot be directly equated with modern anti-ferroptosis therapy. Drugs such as DFO and DFP were initially developed primarily to reduce iron overload and mitigate iron-related oxidative stress. Some early clinical explorations of DFO were even conducted before the concept of ferroptosis was proposed. Therefore, they can only serve as historical evidence of iron homeostasis intervention in AD, and cannot constitute proof that targeted ferroptosis therapy has achieved clear clinical efficacy. Recent DFP research also shows that although it can reduce hippocampal iron load, it does not improve cognitive function in patients with mild AD, but is instead associated with accelerated cognitive decline and an increased risk of neutropenia, suggesting that simply reducing brain iron load is not equivalent to effectively inhibiting ferroptosis.

**FIGURE 2:**
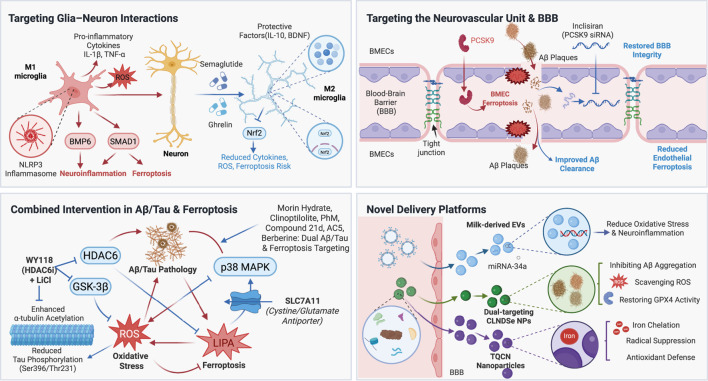
Multi-cellular interaction and multi-pathway synergistic intervention strategy targeting ferroptosis in AD. In Alzheimer’s disease, the anti-ferroptosis intervention framework based on intercellular interactions and multi-pathological pathway integration primarily includes regulation of glia-neuron interactions, protection of the neurovascular unit/blood-brain barrier, synergistic intervention in Aβ/Tau pathology, and novel brain-targeted delivery strategies. Relevant interventions can mitigate neuroinflammation, oxidative stress, endothelial cell damage, and lipid peroxidation accumulation by modulating key pathways such as NLRP3–Nrf2, BMP6/SMAD1, and PCSK9, thereby reducing neuronal susceptibility to ferroptosis. Meanwhile, multi-target small molecules, combined interventions, and nano/extracellular vesicle delivery platforms can further enhance intracerebral delivery efficiency and synergistically improve Aβ aggregation, abnormal Tau phosphorylation, iron homeostasis dysregulation, and impaired antioxidant defenses. This figure highlights that multicellular interactions and multi-pathway synergistic regulation may provide more translationally promising disease-modifying strategies for AD ferroptosis therapy.

Second, drug delivery, specificity, and long-term safety remain key obstacles. Taking DFO as an example, its systemic administration is limited by insufficient BBB permeability and peripheral adverse reactions; although intranasal-brain delivery and other methods can improve central delivery efficiency, their brain distribution, effective exposure concentration, and long-term safety still need to be verified. Meanwhile, iron homeostasis-related molecules are involved in normal neuronal metabolism, mitochondrial function, myelin maintenance, and synaptic activity, and broad-spectrum or non-selective interventions may produce off-target effects. Future research should avoid simply equating “reducing iron load” with “effective anti-ferroptosis.” Instead, it should conduct mechanistic stratification based on iron deposition imaging, lipid peroxidation markers, GSH/GPX4 defense status, Aβ/Tau pathological burden, and inflammatory characteristics. Through precise delivery and well-defined mechanism-based clinical trials, the true value of anti-ferroptosis strategies in disease-modifying therapy for Alzheimer’s disease should be evaluated (Figure 2).

## 3 Cuproptosis

### 3.1 Mechanisms of cuproptosis

Cuproptosis is a recently discovered form of regulated cell death dependent on copper. Its basic mechanism is as follows: excess intracellular copper is reduced by FDX1, subsequently binding to lipoylated TCA cycle proteins such as DLAT and DLST, leading to the aggregation of lipoylated proteins, loss of iron-sulfur cluster proteins, proteotoxic stress, and impaired mitochondrial respiratory function (Tsvetkov et al., 2022). In the pathological context of AD, the disruption of copper homeostasis should not be understood in isolation, but rather in conjunction with disturbances in brain metal homeostasis and mitochondrial metabolic vulnerability. The AD brain is frequently accompanied by abnormalities in the homeostasis of metal ions including copper, zinc, and iron. Both copper and zinc can bind to Aβ and affect its aggregation conformation, deposition, and synaptic toxicity in different ways, with copper also promoting ROS generation through redox cycling (An et al., 2022); abnormal iron homeostasis can further amplify oxidative damage via Fenton reaction and lipid peroxidation. Meanwhile, the loss of Fe–S cluster proteins in the cuproptosis mechanism may further affect mitochondrial electron transport and energy metabolism (Read et al., 2021). Therefore, in the pathological context of AD, cuproptosis-related neurotoxicity is better understood as the combined outcome of multi-metal homeostasis dysregulation, Aβ metal binding, oxidative stress, and mitochondrial metabolic vulnerability.

### 3.2 Pathological links between cuproptosis and AD

#### 3.2.1 Molecular mechanisms of abnormal copper accumulation in AD

Imbalances in brain copper homeostasis are regarded as a key pathological basis for the onset and progression of AD. The basal ganglia and hippocampus are the primary regions for copper distribution and accumulation in the brain, and hippocampal neurons are highly dependent on copper (Desai and Kaler, 2008). As an essential cofactor, copper facilitates key processes such as neurotransmitter synthesis, synaptic transmission, and the maintenance of synaptic plasticity (Gaier et al., 2013). Once copper homeostasis is disrupted, the abnormal accumulation of copper in cognition-related brain regions, such as the hippocampus, becomes a neurotoxic factor. Numerous studies have demonstrated that excessive copper intake and elevated peripheral blood copper levels are significantly associated with cognitive decline (Squitti et al., 2021; Morris et al., 2006). Animal models have further demonstrated that long-term exposure to a copper-rich environment leads to a marked increase in copper content within the hippocampus of C57BL/6J mice. This is accompanied by cuproptosis-like alterations, including upregulation of CTR1, increased DLAT activity, and reduced expression of Fe-S cluster-associated proteins, suggesting that copper overload may mediate neurotoxicity by inducing mitochondrial metabolic damage (Zhang et al., 2023). Furthermore, in a cholesterol-fed rabbit model, it was found that even in a low-concentration copper environment, abnormal amyloid deposition in the hippocampus and temporal cortex was promoted, causing significant impairment of learning and memory abilities. This suggests that abnormal copper accumulation may accelerate the pathological progression of AD (Sparks and Schreurs, 2003). Abnormal brain copper accumulation is not only due to environmental or metabolic factors. Genetic factors may also play a key role. For example, the K832R and R952K polymorphisms of the copper-transport gene ATP7B are associated with an increased AD risk. Researchers have found that the R allele of K832R and the K allele of R952K are both identified as risk alleles. Haplotype analysis further suggests that the R832/K952 haplotype may increase the risk of developing AD, while the K832/R952 haplotype may exert a protective effect (Bucossi et al., 2012). As ATP7B is a key copper-transporting ATPase responsible for maintaining copper homeostasis, its genetic variants may ultimately lead to an imbalance in intracellular copper homeostasis and neurodegenerative pathology by altering copper transport efficiency, elevating free copper levels, and exacerbating oxidative stress.

Recent studies have indicated that the upstream regulatory processes of cuproptosis involve synergistic interactions between RNA-binding proteins and epigenetic modifications. Wang et al. found that the expression levels of the RNA-binding protein polypyrimidine tract-binding protein 1 (PTBP1) were significantly elevated in peripheral blood samples from patients with insomnia/senile dementia (ISD)/AD, as well as in mouse models and *in vitro* neuronal cell models, and that this was closely associated with cognitive decline. At the molecular level, METTL3-mediated m6A modification enhances the stability of PTBP1. PTBP1 subsequently upregulates the expression of SLC31A1 by inhibiting its ubiquitination, ultimately promoting copper uptake and inducing copper-induced neuronal death (Wang et al., 2025b). The aforementioned studies elucidate the upstream regulatory mechanisms of enhanced copper uptake; however, elevated intracellular copper ion levels represent merely the initiating event triggering neuronal damage, and further clarification is required regarding how this specifically activates downstream death pathways. Furthermore, FDX1, a key molecule in cuproptosis, is similarly significantly elevated in peripheral blood samples from AD patients and in in vitro neuronal models, indicating that abnormal copper accumulation is closely associated with the activation of the cuproptosis pathway in AD (Chen et al., 2024).

#### 3.2.2 Cuproptosis in Aβ/tau pathology amplification

Copper homeostasis imbalance can promote the progression of classic AD pathology at multiple levels and forms a mutually amplifying vicious cycle with Aβ and tau protein abnormalities. With regard to Aβ, copper is a key driver promoting its abnormal aggregation and enhanced toxicity. Cu^2+^ forms stable coordination bonds with the N-terminus and histidine residues of Aβ, enhancing peptide-peptide interactions in the early stages of aggregation, thereby promoting protein misfolding, dimerization, and fibril formation (Syme et al., 2004). Furthermore, copper homeostasis disruption can exacerbate the pathological process by influencing Aβ generation and clearance: on the one hand, in animal models, copper exposure regulates the translation and processing of APP in the brains of 3xTg-AD mice and promotes Aβ generation by activating the BACE1-related amyloid pathway (Kitazawa et al., 2009); on the other hand, copper accumulation in cerebral blood vessels and capillaries downregulates the expression of low-density lipoprotein receptor-related protein 1 (LRP1) in the brains of mouse models, which is closely associated with elevated Aβ levels in the brain, suggesting that copper imbalance may lead to persistent accumulation of Aβ in the brain by inhibiting LRP1-mediated Aβ clearance pathways (Singh et al., 2013). Further studies have confirmed that long-term copper exposure not only exacerbates Aβ deposition but also synergistically promotes the formation of senile plaques under high-cholesterol conditions, leading to cognitive impairment (Sparks and Schreurs, 2003; Kitazawa et al., 2009). Furthermore, *in vitro* mechanistic studies have shown that even at sub-stoichiometric levels, Cu^2+^ can significantly accelerate the aggregation kinetics of Aβ and enhance its cytotoxicity, suggesting that even a slight copper imbalance can exacerbate the pathological burden of Aβ (Sarell et al., 2010).

In addition to promoting Aβ aggregation, copper can also significantly enhance its neurotoxicity. A recent dynamic imaging study using a Cu^+^/Cu^2+^-specific DNAzyme fluorescent probe revealed that Aβ oligomerization promotes copper accumulation in neuronal cells, accompanied by elevated Cu^+^ concentrations, increased ROS levels, and the occurrence of FDX1-mediated cuproptosis (Shao et al., 2026). Furthermore, Cu^+^ chelation or FDX1 gene knockdown significantly inhibited neuronal death, whereas the removal of ROS alone only partially alleviated the damage (Chen et al., 2024). This suggests that the core mechanism of Aβ-associated copper neurotoxicity is not simply oxidative stress, but is more likely related to the activation of the FDX1-dependent cuproptosis program against a background of elevated Cu^+^ concentrations. Concurrently, cellular experiments indicate that in neuronal cell models such as SH-SY5Y and HT-22, copper overload not only significantly promotes Aβ oligomerization and its cytotoxicity but also exacerbates neuronal damage by activating the NLRP3/caspase-1/Gasdermin D pathway (Squitti et al., 2021; Zhu et al., 2024). Mechanistic studies have shown that Aβ can bind to Cu^+^, which possesses greater redox activity, to form redox-active copper-Aβ complexes, thereby further increasing ROS production and exacerbating oxidative stress (Guilloreau et al., 2007). Consequently, the interaction between copper and Aβ not only promotes abnormal aggregation but also exerts a dual effect of promoting oxidative toxicity, ultimately leading to neuronal dysfunction and cell death.

Tau protein is also a key target of copper. *In vitro* studies have shown that copper ions can bind to the microtubule-binding repeat regions of tau protein (particularly the R1, R3, and R1-R4 regions), inducing conformational changes and promoting dimerization and fibrillar aggregation (Ma et al., 2005; Ahmadi et al., 2019). In addition to directly promoting aggregation, the copper-tau complex also enhances its redox activity, accelerating substrate oxidation and protein modification, thereby amplifying the toxic effects of tau protein (Bacchella et al., 2020). It is worth noting that, following binding of Cu^2+^ to the tau repeat regions, cysteine-mediated redox reactions promote the formation of disulfide bonds, thereby driving tau dimerization and subsequent aggregation; common antioxidants such as GSH and ascorbic acid have limited inhibitory effects on this process (Ahmadi et al., 2019).

In summary, copper homeostasis imbalance plays a multifaceted promotional role in the pathological progression of AD by promoting the abnormal aggregation of Aβ/tau and enhancing their oxidative toxicity.

#### 3.2.3 Copper-mediated neuroinflammation and oxidative stress

Copper homeostasis imbalance not only directly causes neuronal metabolic damage but may also contribute to the progression of AD by exacerbating neuroinflammation, with microglia likely serving as key effector cells. Studies have shown that in the TgCRND8 transgenic mouse model, the expression of the copper transporter ATP7A is significantly elevated in activated microglia and specifically accumulates around Aβ plaques, suggesting a marked remodeling of copper homeostasis in microglia in AD (Zheng et al., 2010). Chronic copper exposure experiments induce a shift in microglial phenotype towards a degenerative and pro-inflammatory state in both wild-type and J20 model mice, accompanied by cognitive decline. Mechanistic studies have revealed that copper overload significantly enhances the inflammatory response of microglia to Aβ, manifested specifically by abnormal copper accumulation in mitochondria, exacerbated mitochondrial oxidative stress, and activation of the NLRP3 inflammasome (Lim et al., 2020) Furthermore, in cellular models, copper overload can also impair the ability of microglia to phagocytose and clear Aβ by downregulating ATP-binding cassette subfamily A member 7 (ABCA7) expression. These findings suggest that copper overload not only enhances inflammatory signaling but also impairs the protective clearance function of microglia, thereby creating a positive feedback loop of increased Aβ deposition and exacerbated neuroinflammation.

In addition to microglia, astrocytes may also be a key cellular node linking copper homeostasis imbalance with neuroinflammation, oxidative stress, and impaired Aβ clearance in AD. Under normal conditions, astrocytes participate in copper buffering in the brain through systems such as copper transport, glutathione, and metallothioneins, thereby maintaining the stability of the neural microenvironment (Dringen et al., 2013). *In vitro* studies have shown that CuCl_2_ treatment reduces the survival rate of primary astrocytes, intracellular levels of reduced glutathione, and glutathione reductase activity, and increases nitric oxide release, suggesting that copper overload can weaken the antioxidant defense of astrocytes and induce inflammation-related responses (Hu et al., 2016). Further copper ionophore model studies have found that Cu^2+^/elesclomol treatment can increase the lipid peroxidation marker 4-HNE in astrocytes, and antioxidants can partially alleviate cell death, indicating that oxidative stress and lipid peroxidation may be involved in copper-induced astrocyte injury (Ga et al., 2023).

Meanwhile, astrocytes themselves participate in Aβ uptake and degradation, and their clearance function depends on the LRP1/ApoE axis, the endocytosis-lysosomal system, and Aβ-degrading enzymes such as NEP. Liu et al. showed that astrocytic LRP1 deficiency reduces Aβ uptake and degradation, and exacerbates cerebral Aβ deposition in APP/PS1 mice, suggesting that astrocytic LRP1 is an important molecule for maintaining Aβ clearance. Additionally, copper exposure can downregulate LRP1 in the neurovascular unit and impair the Aβ clearance pathway. Copper can also promote the degradation of NEP protein and reduce its enzymatic activity, thereby further weakening Aβ degradation ability (Liu et al., 2017). Based on these findings, copper homeostasis imbalance may disrupt the protective clearance function of astrocytes by inducing oxidative damage, impairing the LRP1/NEP-related Aβ clearance pathway, and promoting glial cell inflammatory responses. Consequently, the Aβ-copper complex can promote ROS generation and glial cell activation, while impaired Aβ clearance capability further exacerbates Aβ deposition, ultimately forming a vicious cycle where copper homeostasis dysregulation, Aβ clearance impairment, oxidative stress, and neuroinflammation mutually reinforce each other.

In addition to endogenous pathological factors, environmental exposures may also promote copper-induced neuronal death by disrupting copper homeostasis and inducing oxidative stress. Recent research findings indicate that polystyrene nanoplastics (PS-NPs) are capable of crossing the BBB in model mice and accumulating in the prefrontal cortex, ultimately leading to impairments in learning, memory, and executive function. From a mechanistic perspective, exposure to PS-NPs significantly increases copper levels within brain tissue and neurons, while also triggering a series of subsequent changes. These changes specifically include upregulation of SLC31A1 expression, increased levels of FDX1, abnormal aggregation of DLAT, elevated heat shock protein 70 (HSP70) levels, and marked damage to mitochondrial structure, while decreased GSH and superoxide dismutase (SOD) levels indicate enhanced oxidative stress. Notably, intervention with N-acetylcysteine (NAC) effectively alleviates pathological damage and improves cognitive function, while the ERK inhibitor PD98059 also mitigates reduced cellular activity and the abnormal expression of cuproptosis-related molecules. These findings suggest that the ERK-MAPK signaling pathway, activated by oxidative stress, is likely involved in this pathological process (Chen et al., 2025).

**FIGURE 3:**
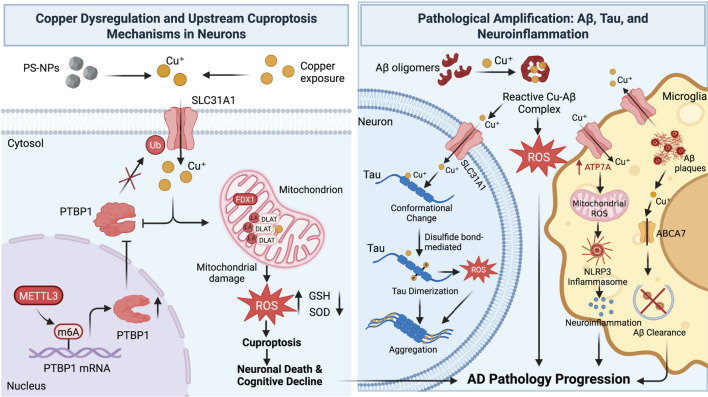
Pathological network of cuproptosis in Alzheimer’s disease. The key pathological network of copper homeostasis imbalance and cuproptosis activation in AD includes the m6A–PTBP1–SLC31A1 axis-mediated enhancement of copper uptake, as well as FDX1-dependent aggregation of mitochondrial lipoylated proteins and disruption of iron-sulfur cluster protein homeostasis. Abnormal copper accumulation can couple with the core pathologies of Aβ/Tau, promoting Aβ oligomerization, Tau conformational abnormalities, and amplification of redox stress, thereby exacerbating neuronal metabolic dysfunction and cell death. Meanwhile, copper overload can drive copper homeostasis remodeling in microglia, NLRP3 inflammasome activation, and impaired Aβ clearance, forming a positive feedback loop among protein deposition, neuroinflammation, and mitochondrial damage. In addition, environmental exposure factors such as polystyrene nanoplastics may further increase the susceptibility of AD neurons to cuproptosis by elevating cerebral copper load, inducing abnormal expression of SLC31A1/FDX1, and promoting DLAT aggregation.

Furthermore, the abnormal changes in key cuproptosis molecules further suggest a close link with immune dysregulation. Studies indicate that FDX1 expression levels show a significant upward trend in the peripheral blood of AD patients and in Aβ-induced neuronal models. Downregulation of FDX1 expression reduces the acetylation levels of DLAT and DLST, while simultaneously alleviating the accumulation of mitochondrial ROS, thereby improving cellular viability. The study also found that the enrichment of γδ-T lymphocytes in the peripheral blood of AD patients is increased, and that FDX1 expression levels are significantly correlated with the infiltration of various immune cells (Chen et al., 2024). Furthermore, multi-omics analysis revealed that AD can be classified into distinct molecular subtypes based on the expression patterns of cuproptosis-associated genes, with these subtypes exhibiting significant differences in the extent of immune infiltration, immune cell composition, and the activation of inflammation-related pathways (Lai et al., 2022). Subtypes with high expression of cuproptosis-associated genes are typically associated with stronger immune cell infiltration and more active inflammatory responses (Nie et al., 2023). Single-cell studies have also revealed that certain cuproptosis-associated signature genes are primarily enriched in peripheral B cells, natural killer (NK) cells, and CD4^+^ T cells, and may participate in intercellular communication via the macrophage inhibitory factor (MIF) signaling pathway (Wang et al., 2025c). Taken together, cuproptosis may not only contribute to mitochondrial metabolic dysfunction within neurons but may also exacerbate pathological changes in the nervous system by reshaping the immune microenvironment (Figure 3).

### 3.3 Therapeutic strategies targeting cuproptosis in AD

#### 3.3.1 Copper chelators

Copper chelators represent an early strategy explored in AD metal homeostasis intervention studies, primarily functioning to bind abnormal free or pathological copper ions, thereby attenuating Cu-Aβ complex-induced Aβ aggregation, oxidative stress, and neurotoxicity. Similarly, the majority of these studies were conducted before the establishment of the cuproptosis mechanism framework and thus cannot be equated with clinical validation targeting the cuproptosis pathway.

Clioquinol is a typical representative of this class of drugs. CQ can interfere with the interaction between metal ions such as copper and zinc and Aβ, thereby reducing metal-dependent Aβ aggregation and oxidative toxicity. In animal models, CQ has demonstrated the potential to reduce cortical and hippocampal Aβ plaque burden and improve cognitive performance; however, in early small-sample clinical trials involving patients with AD, its benefits were primarily observed in the subgroup of patients with moderate-to-severe AD, and its overall efficacy remained unclear (Ritchie et al., 2003). Furthermore, the further development of CQ has been limited by factors such as toxicity risks and insufficient clinical evidence, and thus it has not yet become a mature therapeutic agent for AD. Similarly, tetrathiomolybdate (TTM) primarily alleviates Aβ pathology and inflammatory responses by reducing bioavailable copper levels in the body, but its efficacy may also be influenced by disease stage, leaning more towards potential intervention in early pathological stages (Quinn et al., 2010).

In recent years, further developments in highly selective copper chelators have propelled this strategy towards precision medicine; for example, PA1637 can significantly improve episodic memory in non-transgenic mice with amyloid pathology (Ceccom et al., 2012). The copper-specific chelator TDMQ20 may not only regulate copper homeostasis in the brain but also inhibit oxidative stress reactions catalyzed by Cu-Aβ complexes, and may influence the cholinergic system and synaptic transmission (Zhao et al., 2021). Within the series of imine and quinoline copper chelating molecules L03 to L11, experimental results indicate that L10 exhibits promising preliminary efficacy in a streptozotocin (STZ)-induced rat model of AD; it not only helps restore copper homeostasis in the hippocampus but also alleviates neuroinflammation and oxidative stress, thereby improving spatial memory ability (Camargo et al., 2025). Of particular note is the recently reported bifunctional copper chelator Alz-5; this compound further broadens the scope and applications of copper chelation strategies. In addition to interacting with various Aβ aggregates, it inhibits the formation of Aβ42 fibrils, reduces Aβ42-induced intracellular ROS levels, and reverses abnormal cellular stiffness (Krasnovskaya et al., 2024). Although copper chelators have demonstrated significant improvements in AD symptoms and pathology in most studies, none of them, however, used the core mechanism of cuproptosis as an evaluation endpoint. Therefore, copper chelators should currently be positioned as a candidate strategy for regulating copper homeostasis and metal-Aβ interactions, rather than a validated therapy targeting cuproptosis. Future research on copper chelators should further shift from traditional “broad-spectrum copper scavenging” to “precise regulation of pathological copper.” On one hand, preclinical studies need to systematically evaluate in AD animal models, brain organoids, and human neuronal cell models whether these drugs can affect the core mechanism of cuproptosis; on the other hand, future clinical studies should stratify patients based on brain copper load, non-ceruloplasmin-bound copper, ceruloplasmin levels, Aβ/tau pathological burden, mitochondrial functional status, and inflammatory characteristics, to screen for AD subgroups most likely to benefit from copper homeostasis modulation.

#### 3.3.2 Metal modulators

Unlike traditional copper chelators, metal modulators focus not merely on removing copper ions, but on rebalancing abnormally distributed metals, controlling the movement of copper across cell membranes and its utilization within cells, thereby restoring metal balance to a state close to physiological levels. This approach alleviates the neurotoxicity caused by copper, thereby reducing the risk of cuproptosis. As a typical representative of this class of drugs, the 8-hydroxyquinoline derivative PBT2 demonstrated significant improvements in cognitive function in model mice after 12 weeks of administration. However, in a 12-month clinical trial involving patients with mild AD, while PBT2 played a marked role in reducing Aβ deposits in the brain, there was no significant difference in overall cognitive decline compared to patients who did not receive the drug (Lannfelt et al., 2008). On the other hand, mechanistic studies provide some evidence for its potential therapeutic value: PBT2 facilitates the uptake of extracellular zinc and copper into cells, induces inhibitory phosphorylation of GSK3α/β, and promotes the disassembly of aggregates formed by zinc and Aβ, thereby improving synaptic function and cognitive ability (Crouch et al., 2011). Overall, the findings regarding PBT2 remain controversial across animal models, pathological indicators, and clinical outcomes; this may be related to the heterogeneity of copper homeostasis disturbances at different stages of AD. Future studies should therefore conduct more precise subgroup analyses based on disease stage and copper metabolism characteristics to identify the clinical population that would truly benefit from this treatment. Furthermore, copper-delivery metal modulators have also demonstrated potential for the treatment of AD. For instance, copper bis(thiosemicarbazone) complexes have shown promising efficacy in preclinical studies. Cu(II)(GTSM) enhances intracellular copper utilization, inhibits GSK3β activity, reduces the levels of Aβ trimers and phosphorylated tau protein, and ultimately leads to improved cognitive function (Crouch et al., 2009); while Cu(ATSM) and Cu(DTSM) modulate BBB function, promote Aβ clearance, and alleviate inflammatory damage to the barrier (Pyun et al., 2023; Wasielewska et al., 2024).

#### 3.3.3 Targeting core pathways of cuproptosis

Compared with copper chelation and metal regulation strategies, therapeutic approaches targeting the cuproptosis pathway place greater emphasis on directly intervening at the core execution and regulatory nodes of this process. Current research focuses primarily on targeting the FDX1-mediated copper reduction process, the abnormal aggregation of acetylated TCA enzymes such as DLAT and DLST, and the mitochondrial protein toxic stress associated with cuproptosis. Through a cross-analysis integrating peripheral blood transcriptomic data, neuronal datasets, and cellular experiments, Chen et al. found that FDX1 is a key cuproptosis-associated gene that is significantly upregulated in AD. Further validation in an Aβ25-35-induced SH-SY5Y cell model demonstrated that FDX1 knockdown resulted in reduced acylation levels of DLAT and DLST, as well as decreased accumulation of mitochondrial ROS, thereby mitigating the progression of cell death (Chen et al., 2024).

In addition to directly targeting the mitochondrial execution axis pathway, intervening in upstream pathological cellular communication may also serve as a key strategy for regulating cuproptosis in AD. Ma et al. found that in a 5xFAD mouse model, DLAT protein expression levels showed a significant positive correlation with hippocampal damage and cognitive decline. Further mechanistic studies indicate that microglia-derived exosomes can transport the pyruvate kinase M2 (PKM2) protein into neurons, thereby upregulating DLAT expression and exacerbating copper-induced neuronal death (Ma et al., 2024); conversely, blocking PKM2 transport reduces DLAT levels and alleviates neuronal damage. These findings suggest that cuproptosis in AD is not only caused by intracellular copper overload or mitochondrial metabolic abnormalities, but is also dynamically regulated by pathological communication between glial cells and neurons.

#### 3.3.4 Novel integrated intervention models

In the field of interventions targeting copper-mediated neuronal death in AD, new therapeutic approaches are no longer confined to traditional single-target copper regulation strategies, but are gradually evolving towards multifunctional integration, precision copper scavenging, and platform-driven screening. In terms of multifunctional molecular design, the *Rana* team has developed a class of multifunctional molecules named AST-L1 to AST-L4, which are azo-stilbene-ThT derivatives. These molecules successfully integrate metal-chelating sites with an Aβ-recognition scaffold. These molecules are not only capable of binding copper and zinc ions to regulate Aβ aggregation, but also possess multiple complex functions such as scavenging free radicals, inhibiting cholinesterase, and recognizing amyloid plaques (Rana et al., 2025). Another molecule, known as BDP-CLQ, integrates the copper-chelating activity of CQ with the BODIPY fluorescent scaffold. In addition to inhibiting copper-ion-mediated Aβ aggregation and oxidative damage, it enables optical visualization of Aβ fibrils (Abramchuk et al., 2025). These advances clearly demonstrate that copper-related therapeutic strategies are evolving towards a diagnostic-therapeutic integration model that combines both therapeutic and tracer functions.

Regarding strategies for precise copper regulation, the copper-binding peptide PCu can directly inhibit copper ion-mediated Aβ aggregation and reduce associated toxicity by downregulating BACE1 expression and alleviating oxidative damage (Zhang et al., 2021); the GHK-inspired fluorescent peptide P2N_Gln can preferentially scavenge copper from Aβ-Cu^2+^ complexes, thereby preventing Aβ42 fibrillation while alleviating oxidative/nitrative stress and mitochondrial damage in microglia, demonstrating the potential for synergistic intervention through fine-tuned copper regulation and anti-neuroinflammatory effects (Mandal et al., 2024); meanwhile, the optimized tetrapeptide TP* retains its pathological copper-scavenging capacity while further enhancing stability and barrier-crossing potential under BBB conditions, while minimizing interference with physiological copper-binding systems (López-Guerrero et al., 2025). In addition to peptide molecules, the novel tricoordinating thiosemicarbazone ligand HXE has also demonstrated significant potential for precise copper regulation. By selectively binding to Cu^+^/Cu^2+^, it forms stable complexes in the Aβ-copper pathological environment, thereby inhibiting Cu(Aβ)-mediated ROS generation and correcting abnormal Aβ aggregation (Marinho Barbosa et al., 2026). Unlike traditional broad-spectrum chelation, HXE places greater emphasis on reshaping the pathological copper coordination environment using moderate copper affinity; it can therefore be regarded as one of the representative new strategies in the evolution from “copper removal” to “precision copper regulation” in AD.

**FIGURE 4:**
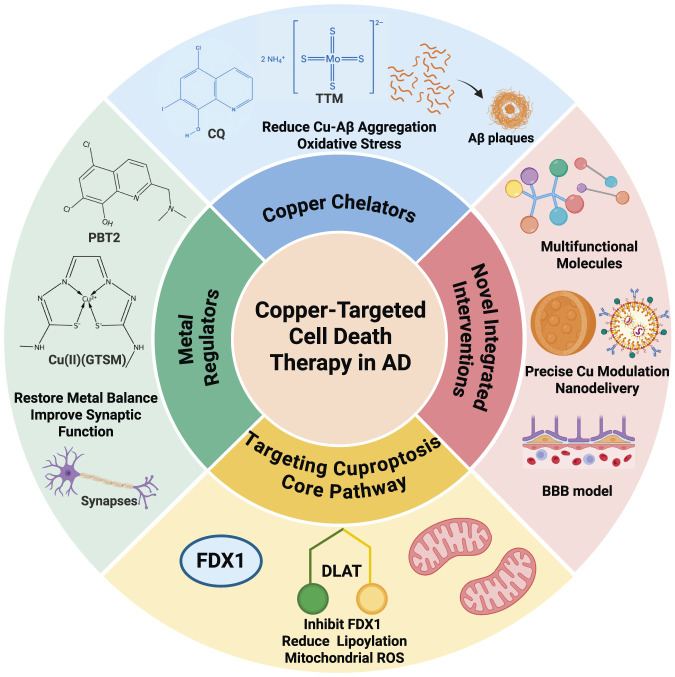
Therapeutic strategies targeting cuproptosis in Alzheimer’s disease. The main intervention directions targeting cuproptosis in AD include copper chelation, metal regulation, blockade of the FDX1–DLAT/DLST execution axis, and novel comprehensive treatment modalities. Copper chelators and metal regulators can mitigate Aβ aggregation and oxidative toxicity by reducing pathological copper load, remodeling copper/zinc distribution, and inhibiting Cu–Aβ complex formation and reactive oxygen species (ROS) generation. Targeting the FDX1–DLAT/DLST axis and microglial exosomal PKM2–DLAT communication can alleviate mitochondrial proteotoxic stress and cuproptosis-related neuronal damage. Multifunctional molecules, precision copper-depleting peptides, nanodelivery systems, and patient-derived blood-brain barrier (BBB) models further advance the development of precise and translational applications for cuproptosis intervention.

In addition to molecular optimization, nanodelivery systems and patient-derived platforms have also provided new translational pathways for this field. Peptide-functionalized gold nanoparticles (PFGNP), utilizing surface-modified functional peptides, can simultaneously inhibit Aβ fibril formation and chelate copper ions, thereby effectively alleviating Aβ-induced toxic damage in SH-SY5Y cells. Furthermore, the patient-derived induced brain endothelial cell (iBEC) model of the BBB provides a screening system more closely aligned with disease characteristics for copper-based drug candidates such as Cu(dtsm), demonstrating that platform-driven screening strategies hold promise for providing new technical support for the translational research of copper-related drugs (Zhou et al., 2022) (Figure 4).

#### 3.3.5 Barriers to clinical translation

The clinical translation of interventions targeting cuproptosis in AD still faces the following barriers: (1) Insufficient effective exposure concentrations in the brain. Both copper-mediated and iron-mediated cell death processes present challenges regarding drug crossing of the BBB. Although copper complexes have been found in recent years to possess some capacity for central nervous system delivery, their delivery efficiency remains poor. For instance, Cu(ATSM) can increase the expression of microvascular P-glycoprotein (P-gp), an ATP-dependent efflux transporter that restricts drug entry into the central nervous system, thereby reducing the BBB transport of its substrates, suggesting that the drug may possess the ability to remodel the BBB transport system; an *in vitro* BBB model constructed from iBECs derived from patients with familial AD identified Cu(dtsm) as possessing superior BBB transport capacity and reducing neuroinflammation. This indicates that overcoming the BBB barrier requires an assessment of drug permeability, interactions between transporters, and local pharmacological effects to achieve precise intervention specifically targeting the pathophysiology of copper-mediated neuronal death in the central nervous system in AD. (2) Insufficient disease staging and patient stratification: Previous studies have shown that copper levels in brain tissue from AD patients may decrease, while serum/plasma copper and non-ceruloplasmin-bound copper (non-Cp Cu) levels increase. Further biomarker research indicates that elevated non-Cp Cu and reduced ceruloplasmin-specific copper are associated with AD risk and can, to some extent, distinguish between AD and non-AD populations. Therefore, future research should focus on developing precision enrichment strategies based on AD disease subtypes, APOE genotype status, and copper metabolism-related biomarkers.

Overall, cuproptosis-related therapies in AD are still in the stage of mechanism translation. Early studies on CQ, PBT2, etc., suggest that regulation of copper/zinc homeostasis and metal-Aβ interaction interventions may improve some pathological processes, but these evidences are not based on the mechanism of cuproptosis and lack systematic validation of core events such as the FDX1-DLAT/DLST axis, lipoylated protein aggregation, Fe-S cluster protein loss, and mitochondrial proteotoxic stress. Therefore, at this stage, relevant strategies should be positioned as candidate interventions for copper homeostasis regulation rather than proven anti-cuproptosis therapies. Future research should conduct precise stratification based on mechanistic biomarkers, disease stages, and copper metabolic characteristics to determine whether cuproptosis-related pathways can become effective targets for disease-modifying therapy in AD.

## 4 Disulfidptosis

### 4.1 Mechanisms of disulfidptosis

In AD, disulfidptosis may be associated with glucose hypometabolism, sulfur metabolic imbalance, and cellular reductive capacity depletion. Under normal conditions, cells maintain cysteine, glutathione (GSH), and disulfide bond homeostasis through the methionine cycle, transsulfuration pathway, and System Xc^−^ (Finkelstein, 2000; Stipanuk, 2004). SLC7A11, as a key subunit of System Xc^−^, mediates the uptake of extracellular cystine while exporting intracellular glutamate. The imported cystine must be reduced to cysteine in a NADPH-dependent manner for GSH synthesis and redox balance maintenance (Koppula et al., 2018; Pannala et al., 2013). However, in the early stage of AD, there is a glucose hypometabolic environment in the brain, and impaired glucose transport and limited pentose phosphate pathway (PPP) can lead to insufficient NADPH production. In SLC7A11-highly expressing cells, sustained cystine uptake further depletes the limited NADPH, leading to abnormal accumulation of cystine and other disulfides, inducing disulfide stress, disrupting the F-actin cytoskeleton, causing cytoskeletal collapse, cell membrane separation, and ultimately resulting in disulfidptosis (Liu et al., 2023).

Furthermore, glutamine metabolism and the serine synthesis pathway may further modulate the susceptibility of AD-related cells to disulfidptosis. SLC7A11-mediated glutamate efflux depletes intracellular glutamate pools, restricting the anaplerotic entry of glutamine into the TCA cycle via glutamate and α-ketoglutarate, thereby reducing metabolic flexibility under conditions of glucose insufficiency (Liu et al., 2023; Shin et al., 2017). The serine synthesis pathway, through one-carbon metabolism, the methionine cycle, and the transsulfuration pathway, influences the homeostasis of homocysteine, cysteine, GSH, and NADPH. Consequently, in the context of glucose hypometabolism in AD, dysfunction of the PPP, restricted glutamine metabolism, and imbalances in serine/one-carbon metabolism may collectively compromise cellular reducing capacity, promote the accumulation of disulfide stress, and link susceptibility to disulfidptosis with redox imbalance, glutamate homeostasis dysregulation, synaptic dysfunction, and neuroinflammation.

### 4.2 Relationship between disulfidptosis and AD

#### 4.2.1 Transcriptomic features of disulfidptosis-related genes in AD

**FIGURE 5:**
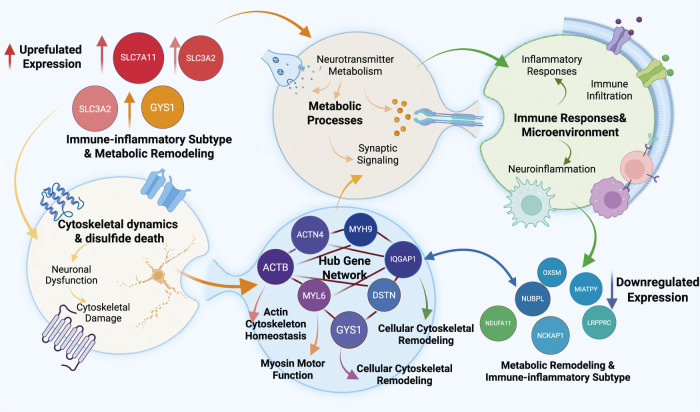
Disulfide death-related molecular networks and pathway interactions in Alzheimer’s disease: from gene expression to neuronal dysfunction. This figure illustrates the potential pathological chain in Alzheimer’s disease (AD), from altered gene expression to neuronal damage, related to molecular abnormalities in disulfidptosis. The aberrant expression of SLC7A11, SLC3A2, GYS1, and actin cytoskeleton-associated molecules such as MYH9, IQGAP1, ACTN4, ACTB, MYL6, and NCKAP1 suggests possible reductive stress imbalance, glucose metabolism remodeling, and disruption of cytoskeletal homeostasis in AD. These molecular alterations may further affect actin remodeling, synaptic signaling, neurotransmitter metabolism, and immune-inflammatory responses, thereby promoting neuronal structural and functional impairments.

Most of the existing evidence linking AD to disulfidptosis stems from research in transcriptomics, bioinformatics, and machine learning; to date, there has been limited direct validation of the underlying mechanisms at the cellular and animal experimental levels. The findings of several existing studies consistently indicate the presence of abnormal molecular expression profiles associated with the disulfidptosis process in AD patients (Ma et al., 2023; Zhu et al., 2023; Huang et al., 2024). Researchers led by Ma screened a set of key hub genes by analyzing GEO cohort data, including molecules such as MYH9, IQGAP1, ACTN4, DSTN, ACTB, MYL6, and GYS1. These genes are primarily involved in closely related biological processes such as the maintenance of actin cytoskeletal homeostasis, myosin movement, and cytoskeletal remodeling. Further subtyping studies revealed significant differences in the degree of immune infiltration among different disulfidptosis-associated subgroups, suggesting that disulfidptosis may contribute to the formation of molecular heterogeneity in AD by causing cytoskeletal damage and reshaping the immune microenvironment (Ma et al., 2023). Similarly, through large-scale transcriptomic analysis, Zhu et al. found that in AD patients, the expression levels of SLC7A11, SLC3A2, and GYS1 were upregulated, while OXSM, NUBPL, NDUFA11, NCKAP1, and LRPPRC were downregulated. Based on these differentially expressed genes, they identified two molecular subtypes: an immune-inflammatory subtype and a metabolic remodeling subtype. This finding suggests that disulfidptosis in AD may involve multiple molecular mechanisms (Zhu et al., 2023). Huang’s research team identified multiple differentially expressed genes associated with disulfidptosis in an independent AD dataset; functional enrichment analysis indicated that these genes are primarily involved in neurotransmitter metabolism, synaptic signaling, and inflammation-related pathways (Huang et al., 2024). These findings further suggest that disulfidptosis-related signaling pathways may be more broadly integrated into the regulation of neuronal dysfunction and inflammatory response networks (Figure 5).

Overall, molecular abnormalities associated with disulfidptosis exist in AD, primarily focusing on several key processes: cysteine metabolism mediated by SLC7A11, energy homeostasis regulation involving GYS1, actin cytoskeleton regulation mediated by NCKAP1/WRC, and the remodeling of the immune microenvironment. These findings suggest that disulfidptosis may participate in the pathogenesis of AD by linking mechanisms such as metabolic dysregulation, cytoskeletal damage, and the amplification of inflammation.

#### 4.2.2 Susceptibility to disulfidptosis in AD

##### 4.2.2.1 Low cerebral glucose metabolism and insufficient reducing power

The occurrence of disulfidptosis depends on restricted glucose supply and insufficient NADPH regeneration; therefore, the persistent brain energy metabolism dysfunction in AD may provide an important metabolic susceptibility background. Minhas et al. (2024) found in animal models that Aβ and tau oligomers can activate indoleamine 2,3-dioxygenase 1 (IDO1) in astrocytes, thereby impairing the metabolic support provided by astrocytes to neurons and inhibiting glucose metabolism in the hippocampus. Furthermore, inhibition or knockout of IDO1 restores glucose metabolism in the hippocampus, enhances glucose entry into glycolysis and the TCA cycle, and improves spatial memory and synaptic plasticity in model mice (Minhas et al., 2024). Meanwhile, Bar et al. (2025) found that promoting glycogenolysis in neurons can divert glucose towards the PPP pathway, thereby increasing the supply of reducing power, alleviating oxidative stress, and mitigating the pathological phenotype of tau protein (Bar et al., 2025). Collectively, these studies indicate that the widespread presence of hypometabolism, insufficient PPP flux, and impaired NADPH supply in AD may weaken the ability of neurons to clear disulfide stress, thereby providing a metabolic basis for disulfidptosis.

##### 4.2.2.2 Weakened Trx–TrxR–NADPH defence line and TXNIP/NLRP3 amplification

The thioredoxin system (Trx/TrxR/NADPH) is a key defense against disulfide stress. Its functional weakening in AD renders cells more vulnerable to disulfidptosis. Within this system, Trx is responsible for reducing cysteine and abnormal protein disulfide bonds, while TrxR relies on NADPH to maintain Trx activity (Qaiser et al., 2024). Once the function of this system is disrupted, the cell’s ability to clear disulfide bond stress is diminished, thereby increasing the risk of cell death. Research by Jia et al. demonstrated that Trx-1 expression levels are reduced in APP/PS1 mouse models and in Aβ-treated cell models. Increasing Trx-1 expression not only restores AMPK-related signaling pathways (Jia et al., 2023) and promotes mitochondrial renewal and function, but also alleviates inflammasome activation by downregulating thioredoxin-interacting protein (TXNIP) expression and inhibiting its binding to NLRP3 (Jia et al., 2025). Furthermore, in non-AD models, inhibition of Trx1 leads to glucose deprivation, subsequently triggering classic disulfidptosis; this process can only be reversed by disulfide bond reducers, while conventional ROS scavengers are ineffective (Tang et al., 2025). The above evidence indicates that persistent impairment of the Trx/TrxR axis and metabolic dysfunction are present in AD. Systemic functional impairment and metabolic disturbances reduce the cell’s ability to clear disulfide stress, promoting the transition of neurons to a state susceptible to disulfidptosis; simultaneously, the Trx-1-TXNIP axis may serve as a crucial bridge linking redox imbalance and inflammatory amplification, suggesting it holds promise as a potential therapeutic target for future interventions against disulfide stress-related damage.

##### 4.2.2.3 Synaptic actin cytoskeletal fragility and NCKAP1/WRC axis abnormalities

Actin cytoskeletal collapse is the most characteristic terminal event of disulfidptosis, and synaptic actin cytoskeletal damage is also strongly suggested in early AD pathology. Haseena et al. found in primary cortical neurons, post-mortem human mild cognitive impairment (MCI)/AD brain tissue, and APP/PS1 mouse models that AD-associated synaptic pathology is accompanied by a significant weakening of PSD-95–actin coupling and a decrease in F-actin levels. More importantly, the F-actin stabilizer jasplakinolide was able to restore synaptic F-actin levels, enhance PSD-95–actin binding, improve glutamate receptor homeostasis, and partially reverse cognitive deficits (Haseena et al., 2024). Furthermore, bioinformatics studies revealed that the molecular profile associated with AD-related synaptotoxicity showed downregulation of NCKAP1 expression. NCKAP1 is a key component of the WAVE regulatory complex (WRC) and plays a crucial regulatory role in maintaining the dynamic equilibrium of actin. Mechanistic studies in non-AD models have revealed that NCKAP1 knockdown partially alleviates the disulfidptosis process induced by glucose deprivation and TrxR1 inhibition, suggesting that the NCKAP1/WRC signaling axis may be involved in cytoskeletal regulation associated with disulfidptosis (Tang et al., 2025). Taking the existing evidence together, the pre-existing synaptic cytoskeletal fragility in AD, coupled with actin dysfunction resulting from abnormalities in the NCKAP1/WRC axis, suggests that actin cytoskeletal abnormalities may constitute a crucial structural basis for the involvement of disulfidptosis in the pathological progression of AD. Future studies need to further validate, in AD models, whether NCKAP1 can serve as a potential therapeutic target for intervening in susceptibility to disulfidptosis.

Overall, existing studies indicate the presence of molecular abnormalities and susceptibility traits associated with disulfidptosis in AD, primarily manifested as dysregulated cysteine metabolism, insufficient energy supply and reducing potential, impaired Trx/TrxR defense system function, and synaptic actin cytoskeletal fragility. However, current evidence in this field is primarily based on transcriptomic analyses, bioinformatic profiling, and mechanistic association studies in non-AD models; direct experimental validation of the typical disulfidptosis process in AD neurons, organoids, and animal models is still lacking. Future research should focus on addressing the following key issues: (1) clarifying the actual occurrence of disulfidptosis in AD; (2) identifying its key regulatory nodes; (3) elucidating the causal relationships between disulfidptosis and Aβ pathology, tau protein abnormalities, synaptic damage, and neuroinflammation. Concurrently, there is a need for a systematic evaluation of the translational medical value of the SLC7A11, Trx/TrxR/TXNIP signaling axis, and the NCKAP1/WRC-actin pathway as potential therapeutic targets.

### 4.3 Translational potential of disulfidptosis in AD

Current research evidence indicates that interventions targeting disulfidptosis have primarily focused on the field of oncology, with the core strategy being to reduce cellular reducing capacity and enhance disulfide stress to induce cell death. In contrast, in the treatment of non-oncological diseases, interventions tend to focus more on utilizing drug-mediated approaches to inhibit disulfidptosis.

Candidate drugs currently supported by experimental evidence primarily include NAC, metformin, 3′-methoxydaidzein (MHD), and vitamin E. Among these, NAC has demonstrated multiple effects in MPTP-induced Parkinson’s disease models, specifically by downregulating SLC7A11 expression and inhibiting the abnormal upregulation of cytoskeletal-related molecules such as ACTN4 and MYL6. These mechanisms suggest that NAC is highly likely to reduce cellular sensitivity to disulfidptosis by alleviating ROS burden, correcting disulfide stress, and simultaneously mitigating cytoskeletal damage (Zhang J. et al., 2025). Metformin has demonstrated direct mitochondrial protective effects in the treatment of ischemic brain injury. Its specific mechanism of action involves stabilizing the Ndufs1-Ndufa11 axis to maintain the function of mitochondrial complex I, thereby improving neuronal survival and electrophysiological function (Cao et al., 2025b). These findings suggest that maintaining mitochondrial homeostasis and restoring the supply of reducing power, thereby inhibiting the activation of the disulfidptosis pathway, may constitute an important intervention strategy. Studies on MHD in ulcerative colitis models indicate that this compound can increase SLC26A2 expression levels and alleviate inflammation and tissue damage. Researchers suggest that it may indirectly inhibit disulfidptosis by modulating the SLC26A2/SLC7A11-related network and alleviating disulfide stress and inflammatory cascades (Yuan et al., 2025). In contrast, research on vitamin E in spinal cord injury has largely relied on bioinformatics analysis and molecular docking predictions (Wang S. et al., 2025). Although it could theoretically inhibit disulfidptosis through antioxidant effects and by regulating IQGAP1-associated cytoskeletal and inflammatory signaling pathways, there is currently a lack of sufficient experimental validation.

At present, research on disulfidptosis in AD remains largely confined to gene screening and the prediction of potential targets. However, studies directly demonstrating, at the cellular or animal level, that drugs exert therapeutic effects by inhibiting disulfidptosis remain scarce. It is worth noting that AD itself is characterized by pathological features such as reduced cerebral glucose metabolism, mitochondrial dysfunction, enhanced oxidative stress, and synaptic cytoskeletal abnormalities. Therefore, conducting drug repositioning studies based on therapeutic strategies involving antioxidant effects, mitochondrial protection, cytoskeletal stabilization, and inflammation buffering may be a key direction for advancing the translation of AD disulfidptosis interventions from mechanistic speculation to therapeutic validation.

### 4.4 Barriers to clinical translation

Currently, research on disulfidptosis in the progression of AD lacks preclinical and mechanistic studies; consequently, the following barriers exist in clinical translation: insufficient mechanistic evidence and target validation. Pioneering studies on the mechanisms of disulfidptosis have primarily focused on tumor cell models. In AD, although there is a highly relevant pathological background involving glucose metabolism, reduced reducing power, and cytoskeletal abnormalities, there is currently a lack of direct evidence to validate the pathological process of disulfidptosis occurring in AD. Consequently, there remains a significant distance to the stage of actual drug development and translational validation. Future work must first validate disulfidptosis disease models in AD-derived cells, organoids, and animal models, while simultaneously establishing a biomarker and multi-omics stratification system to further advance candidate drug screening and central nervous system-targeted delivery.

Ferroptosis, cupraptosis and disulfidptosis all belong to a new class of programmed cell death based on metabolic imbalance; however, the three differ in terms of core triggering factors, key effector molecules, major metabolic pathways, morphological characteristics, as well as the level of evidence and clinical translation progress in Alzheimer’s disease. Existing research suggests that these processes, which correspond to different metabolic pathways, may exhibit cross-coupling within a shared context. Therefore, to clarify the relative positions of these three forms of cell death within the AD pathological network, as well as to determine whether potential links exist and their translational value, it is necessary to conduct a comparative analysis within a unified framework (Table 2).

**TABLE 2:** Comparison of ferroptosis, cuproptosis and disulfidptosis in Alzheimer’s disease: mechanistic features, research evidence and translational implications.

Mode of cell death	Core triggering factors	Key executive molecules	Primary metabolic pathways	Characteristic morphological changes	Strength of direct evidence in AD (human brain/animal/cell)	Diagnostic and therapeutic intervention methods	Clinical evidence and limitations	References
Ferroptosis	Iron homeostasis imbalance, abnormal Fe^2+^ accumulation, lipid peroxidation, and failure of antioxidant systems such as System Xc^−^–GSH–GPX4	ACSL4, SLC7A11, GPX4	System Xc^−^–GSH–GPX4 pathway	Mitochondrial atrophy, increased membrane density, reduction/disappearance of cristae; partial rupture of the outer membrane observed	Human brain tissue: Increased iron deposition in the hippocampus and cortex of AD brains; elevated lipid peroxidation markers such as 4-HNE and MDA; impaired GPX4-related antioxidant defence; accompanied by the progression of Aβ/Tau pathology Animals: Redox imbalance around plaques; downregulation of Fpn and enhanced lipid peroxidation; NOX4-dependent damage in glial cells; ferroptosis contributes to BMEC/perivascular cell damage and BBB disruption; Cells: Aβ-induced detection of decreased GPX4 and elevated ROS and lipid peroxidation; intervention in relevant pathways can partially reverse the damage	Iron homeostasis regulation, anti-lipid peroxidation (DFO, DFP, Fer-1, vitamin E); reconstitute the System Xc^−^–GSH–GPX4/Nrf2 defense line; correct mitochondrial-endoplasmic reticulum-Ca^2+^ homeostasis and metabolic abnormalities; regulate glial cells/neurovascular unit (NVU) and optimize brain-targeted delivery.	Early small-sample studies suggested that DFO may delay cognitive decline; however, in some early-stage AD patients, it was associated with worsening of cognitive function (related to iron homeostasis regulation, not targeting the core mechanism of ferroptosis).	Jiang et al. (2021), Xie et al. (2017), Tan et al. (2021), Kagan et al. (2017), Pope and Dixon (2023), Fujii and Yamada (2023), Stoyanovsky et al. (2019), Li F. J. et al. (2022), Yang et al. (2014), Dixon et al. (2012), Ma et al. (2022), Thorwald et al. (2025), Bao et al. (2021), Park et al. (2021), Chen et al. (2021), Wang et al. (2022), Majerníková et al. (2024), Tang et al. (2024), Zhang H. et al. (2026), Cao Y. et al. (2025), Li J. et al. (2022), Wang et al. (2026), Wang et al. (2023a), Ayton et al. (2025), Zhang et al. (2020), Zilka et al. (2017), Skouta et al. (2014), Deng et al. (2025), Lu Q. et al. (2025), Wang et al. (2025b), Liu et al. (2025), Qian et al. (2026), Xiong et al. (2026a), Li et al. (2025), Li et al. (2024), Peng et al. (2025), Xiong et al. (2026b), Meng et al. (2025), Yong Y. et al. (2024), Baruah et al. (2023), Yong Y. Y. et al. (2024), Gong et al. (2024), Song D. et al. (2025), Han et al. (2026), Zha et al. (2025), Qiu et al. (2023), Zheng et al. (2026), Guo et al. (2025), Mazura et al. (2022), Lu Z. et al. (2025), Abu-Elfotuh et al. (2025), Lv et al. (2025), Kumar et al. (2026), Li et al. (2023), Çelik et al. (2026), Wang et al. (2023b), Liu et al. (2024)
Cuproptosis	Disruption of copper homeostasis, abnormal accumulation of Cu^+^ in mitochondria, abnormal acetylation of TCA cycle proteins, loss of Fe-S cluster proteins, protein toxic stress	FDX1, DLAT, DLST	FDX1–DLAT/DLST–Fe-S cluster pathway	Accumulation of acetylated proteins, loss of Fe-S proteins and impaired mitochondrial respiratory chain	Human brain/peripheral blood samples: Abnormal copper homeostasis; elevated peripheral blood copper levels, increased expression of PTBP1 and FDX1; ATP7B genetic variants associated with AD risk; Animals: Copper exposure leads to copper accumulation in the hippocampus, upregulation of CTR1, increased DLAT, and decreased Fe-S cluster-associated proteins; promotes Aβ deposition, inflammatory activation of microglia, and exacerbates cognitive impairment Cells: Validation that the PTBP1/SLC31A1 axis enhances copper uptake and induces cuproptosis; reduced FDX1 expression alleviates abnormal phospholipid acylation and ROS; Aβ promotes Cu^+^ elevation, induces cuproptosis, and amplifies neuroinflammatory damage	Copper homeostasis regulation and metal homeostasis remodeling (CQ, TTM, PA1637, TDMQ20, L10, Alz-5, PBT2); copper delivery/metal modulation strategies (Cu(II)(GTSM), Cu(ATSM), Cu(DTSM)); targeting the FDX1–DLAT/DLST execution axis and upstream cell communication.	CQ improves cognitive function in patients with moderate-to-severe AD; PBT2 can reduce CSF Aβ42 concentration and improve executive function (related to copper homeostasis regulation, not targeting the core mechanism of cuproptosis).	An et al. (2022), Read et al. (2021), Desai and Kaler (2008), Squitti et al. (2021), Morris et al. (2006), Zhang et al. (2023), Sparks and Schreurs (2003), Bucossi et al. (2012), Wang et al. (2025b), Chen et al. (2024), Syme et al. (2004), Kitazawa et al. (2009), Shao et al. (2026), Zheng et al. (2010), Lim et al. (2020), Chen et al. (2025), Ritchie et al. (2003), Quinn et al. (2010), Ceccom et al. (2012), Zhao et al. (2021), Camargo et al. (2025), Krasnovskaya et al. (2024), Lannfelt et al. (2008), Crouch et al. (2011), Crouch et al. (2009), Pyun et al. (2023), Wasielewska et al. (2024), Ma et al. (2024), Rana et al. (2025), Abramchuk et al. (2025), Zhang et al. (2021), Mandal et al. (2024), López-Guerrero et al. (2025), Marinho Barbosa et al. (2026), Zhou et al. (2022), Dreishpoon et al. (2023)
Disulfidptosis	Glucose deprivation/impaired PPP leading to NADPH depletion; impaired cysteine reduction against a background of high SLC7A11 expression; disulphide stress	SLC7A11, NADPH, F-actin	SLC7A11–NADPH–F-actin pathway	Abnormal disulphidation, contraction and collapse of F-actin, separation of the cell membrane from the cytoskeleton	Human brain tissue: Currently, there is a lack of validation of the typical pathological mechanisms of disulfidptosis; Animals: APP/PS1 models present a susceptible background characterised by impaired PPP and insufficient NADPH supply; direct evidence is still lacking. Cells: Research is currently limited to transcriptomics and bioinformatics studies, with a lack of direct validation	Maintains the PPP–NADPH–Trx/TrxR reduction system; buffers disulphide stress and protects the cytoskeleton; regulates SLC7A11-related susceptibility and metabolic background. Current strategies are primarily indirect protective measures based on mechanistic inferences.	Clinical interventions are primarily based on clinical exploration and still lack clinical evidence.	Koppula et al. (2018), Pannala et al. (2013), Liu et al. (2023), Ma et al. (2023), Zhu et al. (2023), Huang et al. (2024), Minhas et al. (2024), Bar et al. (2025), Qaiser et al. (2024), Jia et al. (2023), Jia et al. (2025), Tang et al. (2025), Haseena et al. (2024), Zhang et al. (2025a), Cao et al. (2025b), Yuan et al. (2025), Wang et al. (2025d)

## 5 Regulatory networks of metabolic cell death

**FIGURE 6:**
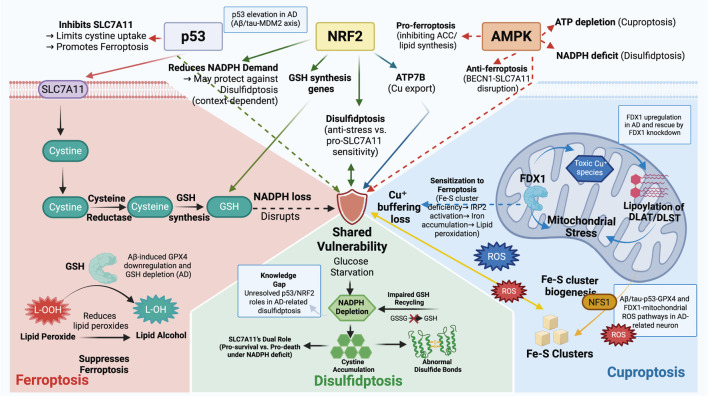
Shared regulatory hubs of metabolic cell death in Alzheimer’s disease. The intersecting regulatory framework of three metabolic cell death pathways in AD primarily includes: the SLC7A11–NADPH–GSH/GPX4 antioxidant axis, the FDX1–DLAT/DLST–Fe-S cluster mitochondrial metabolic axis, and the dynamic transition of upstream regulatory molecular states. Impairment of the SLC7A11–GSH/GPX4 axis can promote lipid peroxidation and ferroptosis, while enhancing disulfide stress and susceptibility to disulfidptosis under NADPH deficiency; Abnormalities in FDX1–DLAT/DLST–Fe-S clusters may induce copper-dependent mitochondrial proteotoxicity and increase ferroptosis risk through disrupted Fe-S cluster homeostasis, remodeled iron metabolism, and ROS accumulation; As upstream regulators, p53, NRF2, and AMPK can dynamically influence the balance and transition between different cell death pathways based on cellular metabolic status, reducing power levels, and Aβ/Tau pathological contex.

AD is not a neurodegenerative disease driven solely by a single mechanism of RCD. Current research findings suggest that the disease is more likely to be triggered by the combined action of multiple pathological factors. These factors specifically include redox homeostasis imbalance, metal ion metabolism disorders, mitochondrial dysfunction, and persistent, escalating neuroinflammatory responses. The combined action of these factors renders neurons vulnerable. Within this pathological microenvironment, multiple metabolic-related cell death pathways are interconnected via shared metabolic nodes and stress signals, ultimately forming a complex cell death regulatory network (Table 3) (Figure 6).

**TABLE 3:** Shared molecular hubs and regulatory crosstalk among metabolic cell death pathways in Alzheimer’s disease.

Name/Hub	Mechanism of action/regulatory mechanism	Associations in AD	References
SLC7A11–NADPH–GSH/GPX4 axis	Ferroptosis: SLC7A11 uptake of cysteine → GSH synthesis → GPX4 reduction of L-OOH, inhibiting lipid peroxidation.	Aβ-induced ferroptosis is often accompanied by reduced GPX4 and GSH depletion. In AD brains, decreased PPP flux and insufficient NADPH supply weaken the antioxidant effects of GSH; in APP/PS1 mouse astrocytes, SLC7A11, GSH and GPX4 are downregulated, accompanied by the activation of ferroptosis;	Dixon et al. (2012), Maimaiti et al. (2024), Baruah et al. (2023), Liu and Gan (2021), Zhang et al. (2025b), Patel et al. (2024), Correas et al. (2024), Ho et al. (2022)
​	Disulfidptosis: Impaired cysteine reduction due to NADPH deficiency → abnormal accumulation of disulphide bonds; high expression of SLC7A11 increases susceptibility to disulfidptosis.	Impaired glucose homeostasis can simultaneously promote disulfidptosis, cuproptosis and ferroptosis. APP/PS1 mice exhibit impaired PPP energy metabolism; enhancing G6PD/PPP may increase NADPH supply and alleviate disulphide stress.	
	Cuproptosis: Decreased GSH weakens Cu^+^ buffering, enhancing cuproptosis.	Aβ aggregation and oxidative stress in the AD brain can lead to neuronal GSH depletion, impairing the cell’s ability to buffer and detoxify free copper	
FDX1–DLAT/DLST–Fe-S cluster axis	Cuproptosis: FDX1 promotes the formation of toxic copper species, damaging the acetylated proteins DLAT/DLST.	Elevated FDX1 levels in AD patients and Aβ models; FDX1 knockdown alleviates DLAT/DLST acetylation abnormalities, reduces mitochondrial ROS, and improves cell viability.	Tsvetkov et al. (2022), Chen et al. (2024), Alvarez et al. (2017), Terzi et al. (2021)
	Ferroptosis: Defects in Fe-S clusters → IRP2-mediated iron starvation response → increased available iron pool → enhanced sensitivity to ferroptosis.		
	Disulfidptosis: It is speculated that this may exert an indirect effect via mitochondrial dysfunction.		
p53	Ferroptosis: Transcriptional repression of SLC7A11 → Weakening of the GSH/GPX4 defence line, enhancing ferroptosis; however, the p53-p21 axis may delay ferroptosis during cysteine deficiency.	Elevated p53 levels in the brains of AD patients (superior temporal gyrus); Aβ42 activates the p53 promoter, whilst tau inhibits p53 degradation; this may link Aβ/Tau pathology to the metabolic death network.	Jiang et al. (2015), Tarangelo et al. (2018), Hooper et al. (2007), Ohyagi et al. (2005), Sola et al. (2023)
	Disulfidptosis: Downregulation of SLC7A11 reduces NADPH consumption, theoretically lowering susceptibility to disulfidptosis (no direct evidence).		
NRF2	Ferroptosis: Induction of SLC7A11, GCLC and others enhances the GSH-GPX4 defence line, thereby inhibiting ferroptosis.	The direction of action depends on the cellular metabolic background, redox status and SLC7A11 expression levels. Abnormal NRF2 expression in AD hippocampal neurons impairs the antioxidant transcriptional response; hippocampal	Livingston et al. (2024), Tang et al. (2025), Uruno et al. (2020), Kanninen et al. (2009), Ramsey et al. (2007), Shi et al. (2025), Tanito et al. (2007), Harvey et al. (2009), Feng et al. (2021), Du et al. (2024)
	Copper-induced cell death: The Keap1/Nrf2/ATP7B axis promotes copper efflux, raising the threshold for copper-induced cell death.		
​	Disulfidptosis: dual action—enhances the thiol reduction system (e.g., TrxR1) to buffer disulphide stress; however, SLC7A11 activation may also increase susceptibility to disulfidptosis.	Nrf2 gene delivery improves spatial learning and memory in APP/PS1 mice; upregulation of Nrf2 suppresses oxidative stress and neuroinflammation, and promotes Aβ clearance	​
AMPK	Ferroptosis: bidirectional — promotes the formation of the BECN1–SLC7A11 complex, enhancing ferroptosis; or inhibits ACC to reduce PUFA synthesis, thereby suppressing ferroptosis.	AD: abnormal accumulation of activated AMPK in the brain occurs in pre-entangled/entangled neurons and around Aβ plaques; Aβ exposure can activate AMPK, which can directly phosphorylate tau; under persistent Aβ stimulation and chronic pathological stress, excessive AMPK activation can exacerbate synaptic plasticity impairments and memory damage	Zimmermann et al. (2020), Thornton et al. (2011), Vingtdeux et al. (2011), Mi et al. (2026), Liu et al. (2022), Lee et al. (2020), Song et al. (2018)
	Cuproptosis: acting as a molecular response to copper-induced energy stress, its activation regulates the extent of cuproptosis and inflammatory signalling.		
	Disulfidptosis: maintains the balance between energy and reducing power under glucose deprivation; promotes disulfidptosis when activation is impaired.		

The SLC7A11–NADPH–GSH axis is precisely a representative regulatory node in this network. Under conditions with sufficient reducing power, SLC7A11 promotes cystine uptake, cysteine production, and GSH synthesis, thereby supporting GPX4 in scavenging lipid peroxides and reducing ferroptosis sensitivity. However, in the context of Alzheimer’s disease-related glucose hypometabolism, insufficient pentose phosphate pathway flux, and impaired NADPH regeneration, sustained cystine uptake further depletes the limited reducing power, leading to disulfide accumulation, disulfide stress, and F-actin cytoskeleton disassembly, thereby increasing susceptibility to disulfidptosis. Although SLC7A11 is not a core molecule of classical cuproptosis, the cysteine/GSH system it maintains can indirectly affect cellular tolerance to copper overload and cuproptosis-related stress through copper chelation, protein sulfhydryl protection, and mitochondrial redox regulation. Therefore, SLC7A11 is not a single protective factor but may be a metabolic switch influenced by nutritional status and reducing power levels, linking ferroptosis, disulfidptosis, and cuproptosis-related damage in AD. The remaining pathway networks are shown in Table 3.

## 6 Humoral biomarkers and patient stratification for metabolic cell death

In recent years, research into the pathological mechanisms of metabolic cell death in AD has deepened; clinically detectable, stratifiable, and dynamically monitorable humoral biomarkers remain a key element in advancing related research towards precision diagnosis and treatment. Unlike Aβ- and tau-related biomarkers, metabolic biomarkers complement the pathological mechanisms of AD by addressing redox imbalance, metal homeostasis disruption, and susceptibility to cell death (Table 4).

**TABLE 4:** Humoral biomarkers of metabolic cell death and patient stratification strategies in Alzheimer’s disease.

Candidate biomarkers	Sample type	Associated mode of cell death	Clinical sample validation	Association with the disease	Current status and specificity	References
4-HNE	Peripheral blood	Ferroptosis	4-HNE levels are significantly elevated in AD patients; plasma HNE has a sensitivity of 93.5%, specificity of 75% and an AUC of 0.93	Associated with elevated plasma Aβ42 and reduced total tau, but inconsistent results regarding its association with the MMSE, suggesting it is more indicative of pathological burden than a stable staging marker.	Reflects end-products of lipid peroxidation; associated with AD ferroptosis, lipid peroxidation and pathological burden; however, it is not a marker specific to ferroptosis	Lovell et al. (1997), Rani et al. (2017), McGrath et al. (2001)
MDA	Peripheral blood	Ferroptosis	Plasma MDA is elevated in AD and MCI, with a sensitivity of 80%, specificity of 75% and AUC of 0.87 for the diagnosis of AD	Elevated in both MCI and AD, suggesting that abnormalities may occur in the early stages of the disease, but the association with severity indicators such as the MMSE is inconsistent.	Associated with ferroptosis, oxidative stress and lipid peroxidation in AD; limited specificity for diagnosing AD ferroptosis	McGrath et al. (2001), Zoroddu et al. (2026)
GSH/GSSG ratio	Peripheral blood	Ferroptosis	In AD patients, plasma GSH levels are reduced, GSSG levels are elevated, and the GSH/GSSG ratio is decreased; diagnostic sensitivity for AD is 91.1%, specificity is 97.8%, and AUC is 0.95	Correlated with Aβ42 and total tau levels in AD pathology; the peripheral GSH/GSSG ratio in patients with MCI and AD is significantly lower than in non-AD patients; suggesting early changes on the AD continuum	Reflects impairment of the Xc^−^–GSH axis and redox imbalance; may serve as an auxiliary indicator of potential risk and disease progression	McGrath et al. (2001)
CSF ferritin	Cerebrospinal fluid	Ferroptosis	Baseline CSF ferritin is associated with cognitive decline over 7 years and can predict the conversion of MCI to AD	A humoral biomarker of brain iron load; associated with the risk of AD progression and conversion	A surrogate marker for abnormal brain iron load/iron homeostasis; may indicate a predisposition to ferroptosis and risk of conversion to AD	Ay et al. (2015), Ayton et al. (2023)
SLC7A11	Peripheral blood leukocytes	Ferroptosis; bisulfide-mediated cell death	Elevated SLC7A11 mRNA expression in peripheral white blood cells of AD patients; diagnostic cutoff value for AD is 12.185, sensitivity 0.954, specificity 0.523, AUC 0.803	Enhanced expression may amplify the toxicity of pathological Aβ in AD; demonstrates the ability to distinguish between AD and non-AD; has not yet been consistently associated with disease severity	System Xc^−^ core subunit; serves as a shared hub molecule for ferroptosis and disulfidptosis; currently holds potential for disease diagnosis and mechanism stratification, but specificity is generally low and further validation is required	Lane and Lin (2023)
Ceruloplasmin	Cerebrospinal fluid	Primarily copper-mediated, with some iron-mediated components	Elevated ceruloplasmin levels in MCI patients with Aβ pathology predict faster cognitive decline and ventricular enlargement	Ceruloplasmin is a molecule linking copper metabolism abnormalities, impaired iron efflux and oxidative stress; elevated levels are significantly associated with disease progression in the AD subgroup of MCI patients	It serves as a supplementary marker of the copper-iron redox and transport system; it holds potential for prognosis and stratification in Aβ-positive MCI populations, but is not a direct executor marker of cuproptosis	Diouf et al. (2020), Squitti et al. (2023)
FDX1	Peripheral blood	cuproptosis	FDX1 mRNA expression is elevated in the peripheral blood of AD patients, and is even higher in APOE ε4/ε4 patients	Knockdown of FDX1 in AD models reduces DLAT/DLST acetylation and attenuates mitochondrial ROS; FDX1 is also associated with alterations in peripheral immune cell infiltration in AD	A key molecular component of cuproptosis; it holds potential for distinguishing AD subtypes and stratifying mechanisms, but remains a candidate biomarker in the early stages of validation.	Chen et al. (2024)

Based on existing research evidence, humoral biomarkers associated with metabolic cell death in AD have preliminarily shown a trend shifting from ‘downstream damage’ to ‘proximal mechanism-related markers’, allowing for the preliminary proposal of a stratification framework comprising ferroptosis-dominant, copper-dominant, and mixed types. The ferroptosis-dominant subtype is primarily characterized by elevated 4-HNE and MDA levels, a decreased GSH/GSSG ratio, and may be accompanied by elevated CSF ferritin and abnormal SLC7A11 expression, suggesting enhanced lipid peroxidation, impairment of the GSH/GPX4 defense line, and increased brain iron load. Patients in this category may benefit from interventions such as iron chelation, inhibition of lipid peroxidation, restoration of the upstream ferroptosis pathway (Xc^−^–GSH–GPX4 axis), or enhancement of cellular reducing capacity. In contrast, the copper-dominant subtype is primarily characterized by abnormal copper homeostasis, abnormal CSF ceruloplasmin levels, and elevated FDX1, and these patients are more responsive to interventions targeting copper homeostasis regulation, copper chelation, copper ion redistribution, and the FDX1–DLAT/DLST axis. For the mixed subtype, which exhibits pathological abnormalities associated with both iron and cuproptosis, multi-target combined interventions may be required. Upstream regulatory mechanisms, such as miRNAs, m6A modifications, and RNA-binding proteins (e.g., PTBP1), contribute to the formation of metabolic cell death networks. In the future, this may represent a direction with translational potential, involving the screening of candidate molecules that can be stably detected in blood, CSF, and exosomes, and the evaluation of their clinical value in early screening, mechanism stratification, and efficacy assessment.

A key limitation in the current field of biomarker research is that the correspondence between these biomarkers and specific clinical stages of AD remains unclear. Ideally, clinically applicable biomarkers should be able to play a predictive role in the preclinical stage or the mild cognitive impairment (MCI) stage. For example, elevated cerebrospinal fluid ferritin and a decreased plasma GSH/GSSG ratio have shown potential in predicting the conversion from MCI to AD, suggesting that they may be early indicators of ferroptosis susceptibility. In contrast, terminal lipid peroxidation markers such as plasma 4-HNE and MDA, although elevated in AD patients, are typically more strongly correlated with disease severity and pathological burden (Aβ/tau) and may not necessarily be suitable as early predictive biomarkers. For copper metabolism, abnormal cerebrospinal fluid ceruloplasmin appears to predict faster cognitive decline in MCI patients with Aβ pathology, thus potentially serving as a valuable prognostic biomarker in this specific subgroup. However, biomarkers such as FDX1 are still in the early validation stage, and their utility across different disease stages remains unclear. Future longitudinal studies are urgently needed to systematically characterize the temporal dynamics of these biomarkers across the entire AD continuum, from the preclinical stage to severe dementia, thereby clarifying their practical value in early diagnosis, prognosis assessment, and patient stratification.

## 7 Summary and outlook

This paper breaks away from previous review models focusing on single cell death mechanisms, and for the first time integrates ferroptosis, cuproptosis, and disulfidptosis within a common metabolic context, proposing that metabolic cell death in AD should be understood as a ‘metabolic-death network’. Within this network, metal homeostasis disruption, redox imbalance, mitochondrial dysfunction, and glucose metabolism abnormalities are not independent events, but rather collectively create a state of neuronal vulnerability through shared hubs. The AD brain is characterized by persistent metal ion imbalance, lipid peroxidation, abnormalities in the mitochondrial respiratory chain, reduced cerebral glucose metabolism, and chronic inflammation; these factors collectively constitute the basis of metabolic abnormalities. There are key common regulatory nodes between the three modes of cell death: the SLC7A11–NADPH–GSH/GPX4 axis simultaneously influences susceptibility to all three modes, while the FDX1–DLAT/DLST–Fe-S cluster extends the mitochondrial protein toxic stress in cuproptosis to the levels of iron homeostasis and oxidative stress. Upstream factors such as NRF2, p53, and AMPK converge upstream of all three pathways, capable of simultaneously driving multiple metabolic cell death programs. Consequently, while different modes of cell death may exhibit relative dominance in distinct brain regions, cell types, or disease stages, they are driven by the same metabolic network overall, mutually amplifying one another, and jointly contributing to the degenerative pathology of AD neurons. From the perspective of pathological progression, metabolic cell death in AD may follow a sequential activation pattern: in the early stages, it begins with increased susceptibility to ferroptosis, manifested as iron metabolism imbalance, enhanced lipid peroxidation, and impairment of the GPX4 defense line; in the middle stages, as Aβ and tau toxicity intensify and copper homeostasis and mitochondrial function deteriorate, the ferroptosis process gradually amplifies neuronal damage; in the late stage, due to a persistent decline in cerebral glucose metabolism and NADPH depletion, disulfide stress and cytoskeletal damage worsen, and disulfidptosis leads to irreversible neuronal damage. Given these network-based and stage-specific characteristics, therapeutic strategies should shift towards identifying critical windows and patient stratification. The APOE ε4 gene is significantly associated with cerebral iron load and the risk of ferroptosis, and the associated reduction in cerebral glucose metabolism is already evident in the preclinical phase, suggesting that carriers enter a state of metabolic vulnerability earlier. Therefore, interventions targeting ferroptosis should be initiated earlier, with a focus on copper and energy metabolism. In clinical practice, early-stage management should focus primarily on inhibiting ferroptosis and improving metal homeostasis and redox imbalance; in the intermediate stage, further regulation of copper homeostasis and mitochondrial protein toxic stress is required; while in the late stage, the emphasis should be on restoring cellular reducing capacity, protecting NVUs, and providing comprehensive supportive care, thereby achieving stratified and precision-based diagnosis and treatment.

Although relevant research is increasing, several major limitations currently remain. Existing studies largely focus on single pathways and lack an integrated perspective: research on ferroptosis is relatively mature, whereas cuproptosis and disulfidptosis remain primarily confined to transcriptomic analyses and a small number of *in vitro* experiments, with insufficient evidence regarding direct mechanisms. Animal models rely heavily on transgenic and Aβ-induced models, with limited capacity to replicate the complex pathological background of sporadic AD, such as its hypometabolic state and metal homeostasis imbalance; furthermore, there is a lack of an ideal *in vivo* experimental system capable of simultaneously detecting all three forms of cell death. The determination of cell death lacks specificity; existing biomarkers (such as 4-HNE, MDA, GSH, and GPX4) primarily reflect oxidative damage, making it difficult to distinguish between different modes of cell death. Intervention studies remain at the preclinical stage; existing strategies primarily involve natural products, antioxidants, and metal chelators, with a lack of large-scale clinical trial data. Furthermore, limitations of the BBB and risks of peripheral toxicity hinder clinical translation. Future breakthroughs must be advanced on two fronts: key scientific questions and technological innovation. Regarding scientific questions, it is necessary to determine whether there are key hub nodes within the metabolic death network that regulate cell fate transitions; whether these nodes exhibit specificity across different brain regions, cell types, and disease stages; whether the three modes of cell death occur independently or involve sequential activation and mutual conversion; and to identify the molecular switches that trigger the transition of neuronal cells from reversible stress to irreversible death. In terms of technological innovation, spatial transcriptomics and single-cell omics technologies can be utilized to construct spatiotemporal distribution maps of the three modes of cell death; machine learning and artificial intelligence can be applied to biomarker screening, molecular subtype identification, and drug design; *in vivo* dynamic imaging techniques hold promise for real-time monitoring of key indicators such as metal ion dynamics and lipid peroxidation; and brain-like organoids and human 3D model systems can overcome the limitations of traditional models. By integrating these perspectives and technical approaches, research into metabolic cell death in AD is expected to advance towards a precision medicine approach with greater clinical translational value.
